# Supplementary material for: Steric and Electronic Effects in N-Heterocyclic Carbene Gold(III) Complexes: An Experimental and Computational Study
Source: Molecules. 2022 Nov 28;27(23):8289. doi: 10.3390/molecules27238289 (PMC9740751; doi:10.3390/molecules27238289)
Supplement: Supplementary file 1 [file molecules-27-08289-s001.zip › molecules-2050487-supplementary.pdf]

Supporting Information for

Steric and Electronic Effects in N-Heterocyclic Carbene Gold(III) Complexes: An Experimental and Computational Study

Miguel A. Rosero-Mafla [1], Jhon Zapata-Rivera [2], M. Concepción Gimeno [3,] and Renso Visbal [1,4,*]*

[1]Facultad de Ciencias Naturales y Exactas, Departamento de Química, Universidad del Valle, A.A. 25360, Cali, Colombia

[2]Departamento de Química, Facultad de Ciencias, Universidad de los Andes, Cra 1 No 18A – 12, Bogotá 111711, Colombia

[3]Departamento de Química Inorgánica, Instituto de Síntesis Química y Catálisis Homogénea (ISQCH), CSIC-Universidad de Zaragoza, 50009 Zaragoza, Spain

[4]Centro de Excelencia en Nuevos Materiales (CENM), Universidad del Valle, A.A. 25360, Cali, Colombia

Electronic spectra

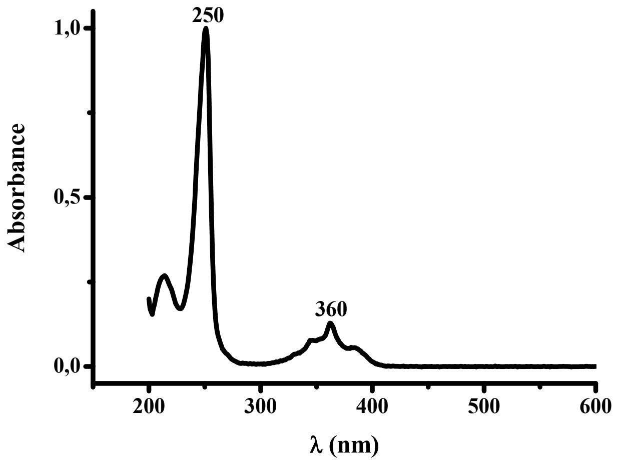

Figure S1. Electronic spectrum of complex **1**.

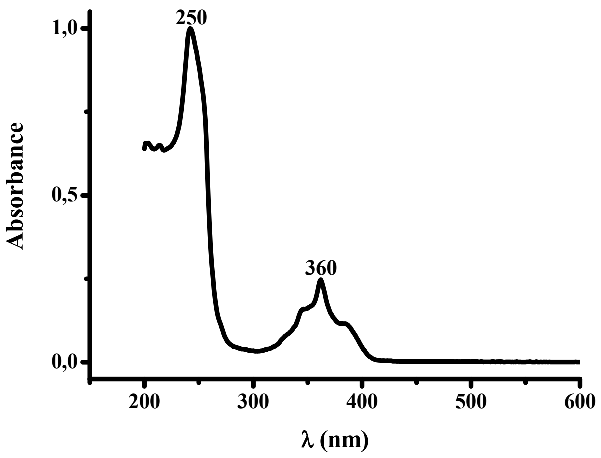

Figure S2. Electronic spectrum of complex **2**.

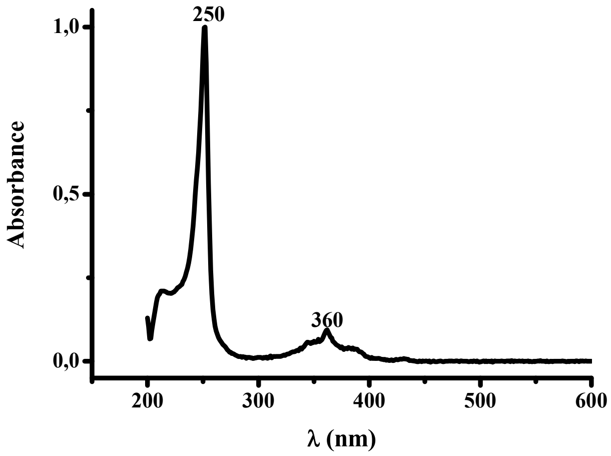

Figure S3. Electronic spectrum of complex **3**.

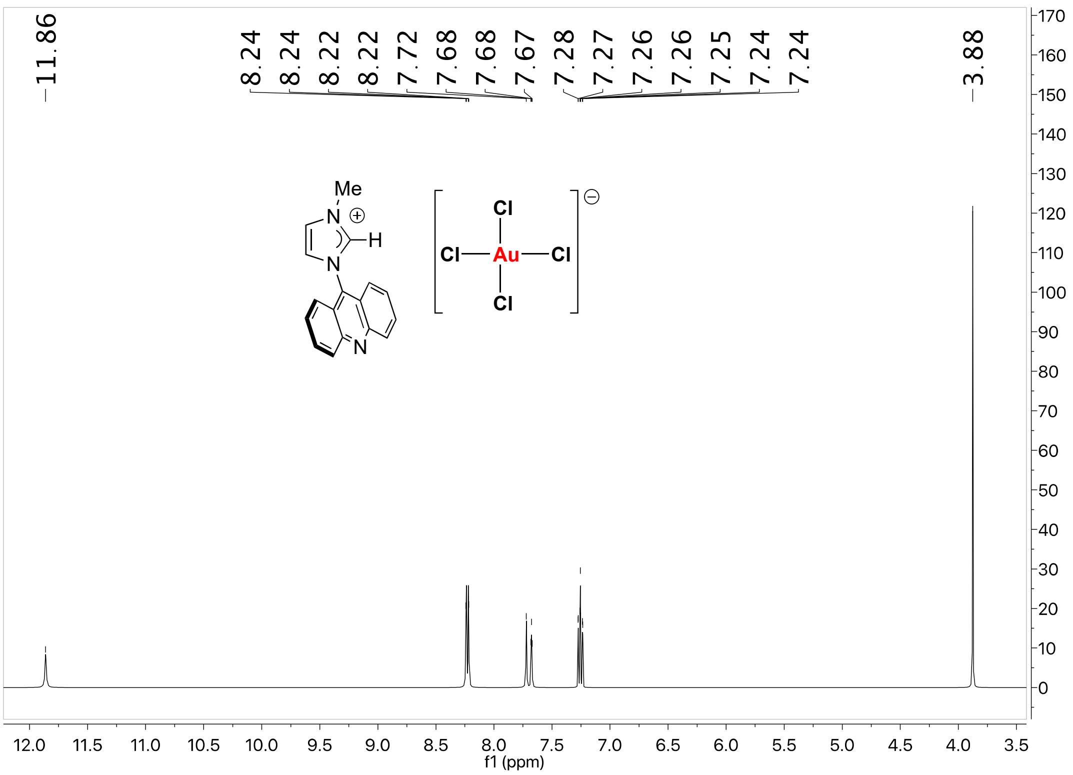

Figure S4. ${}^{1}H$ NMR spectrum of intermediate **1** in DMSO-d^6.

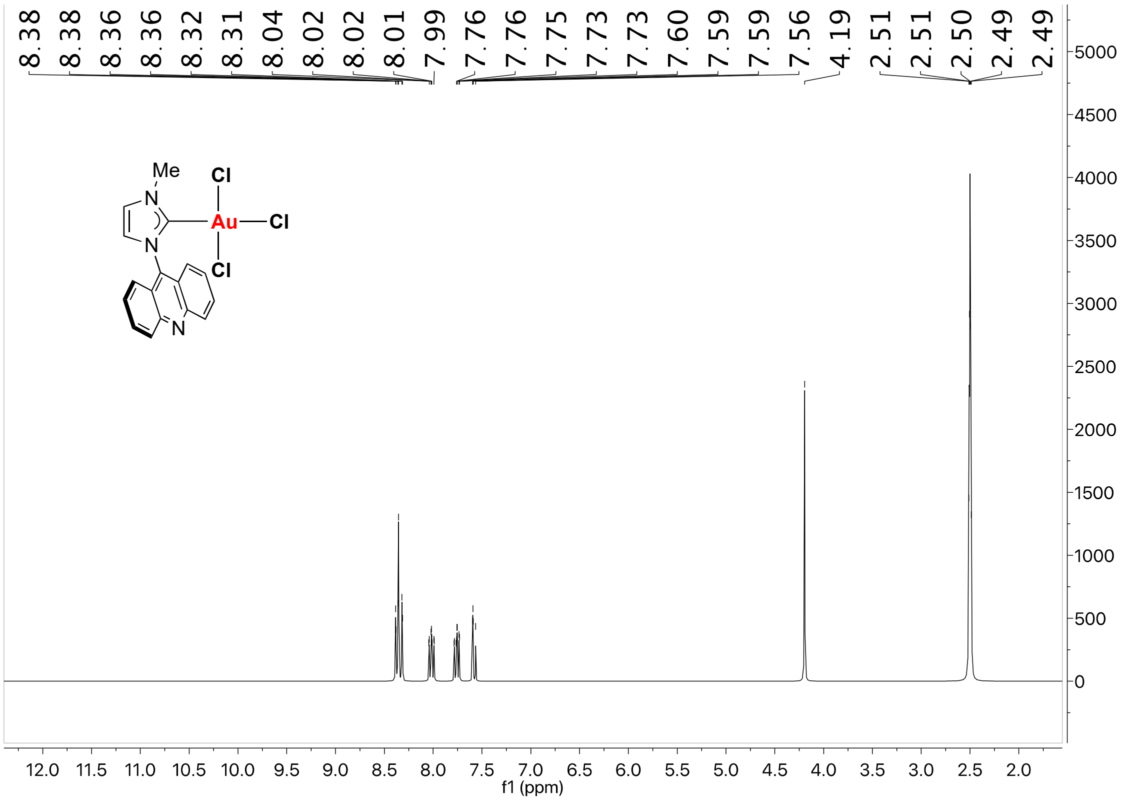

Figure S5. ${}^{1}H$ NMR spectrum of complex **1** in DMSO-d^6.

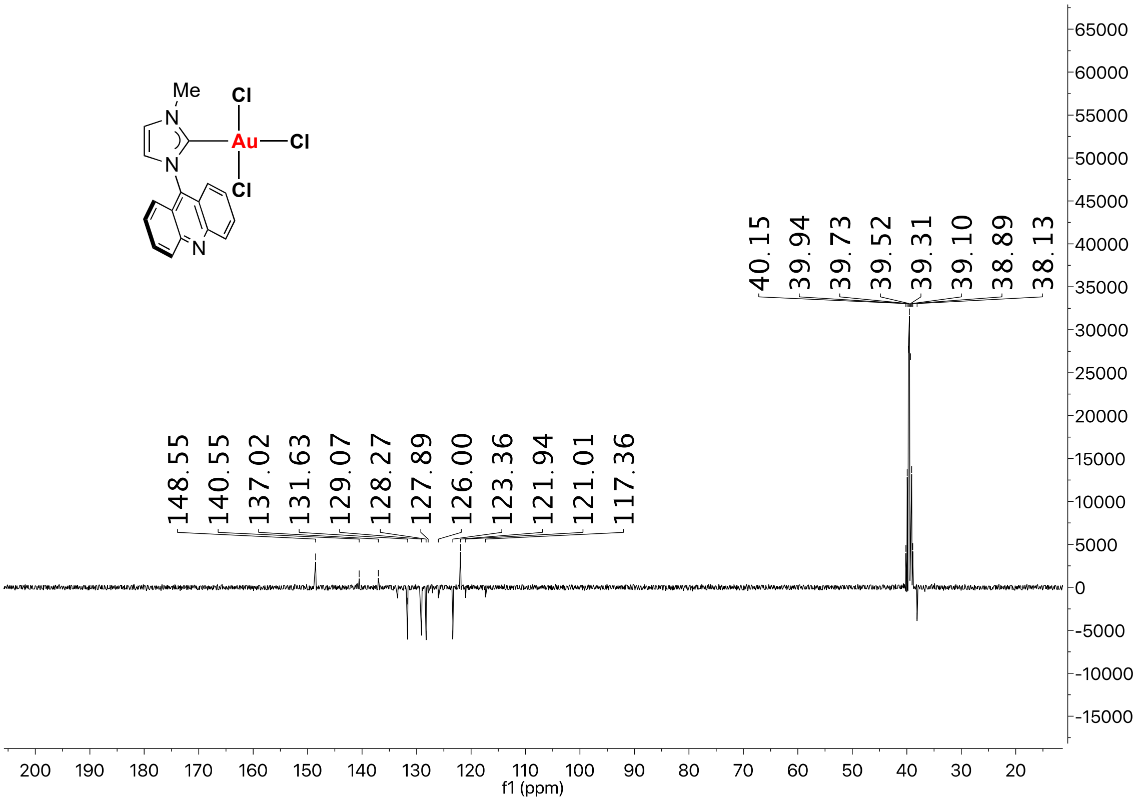

Figure S6. $^{13}C-\{^1H\}$ (APT) NMR spectrum of complex **1** in DMSO-d^6.

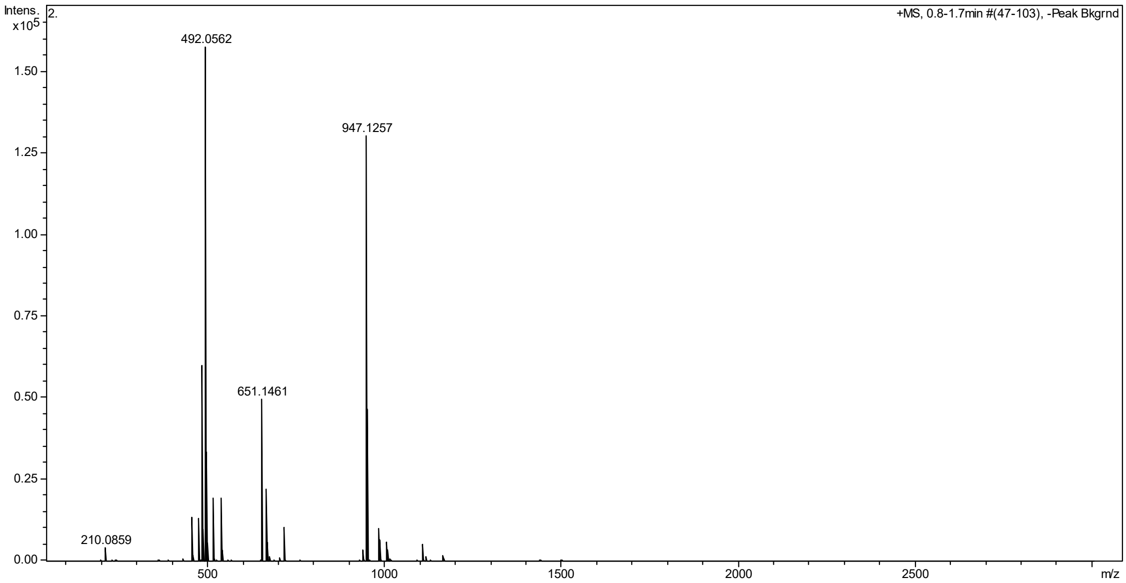

Figure S7. ESI$^+$-MS spectrum of complex **1**.

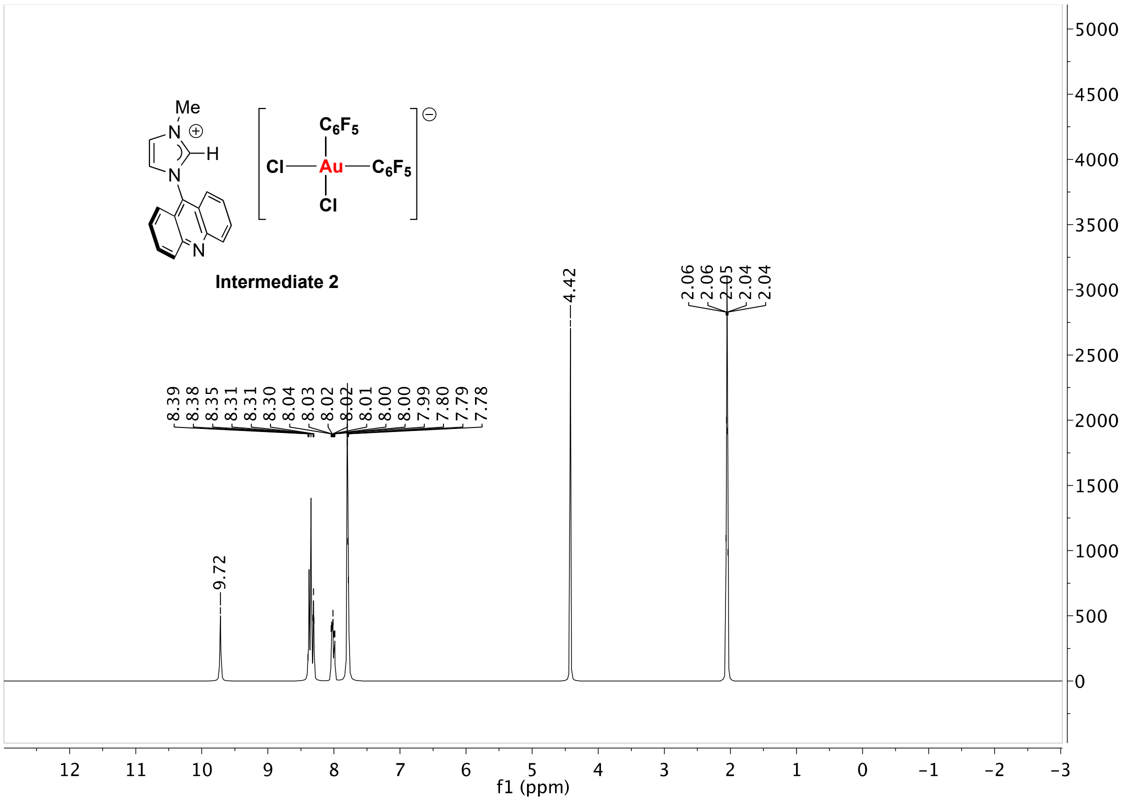

Figure S8. ^{1}H NMR spectrum of intermediate **2** in acetone-d^6.

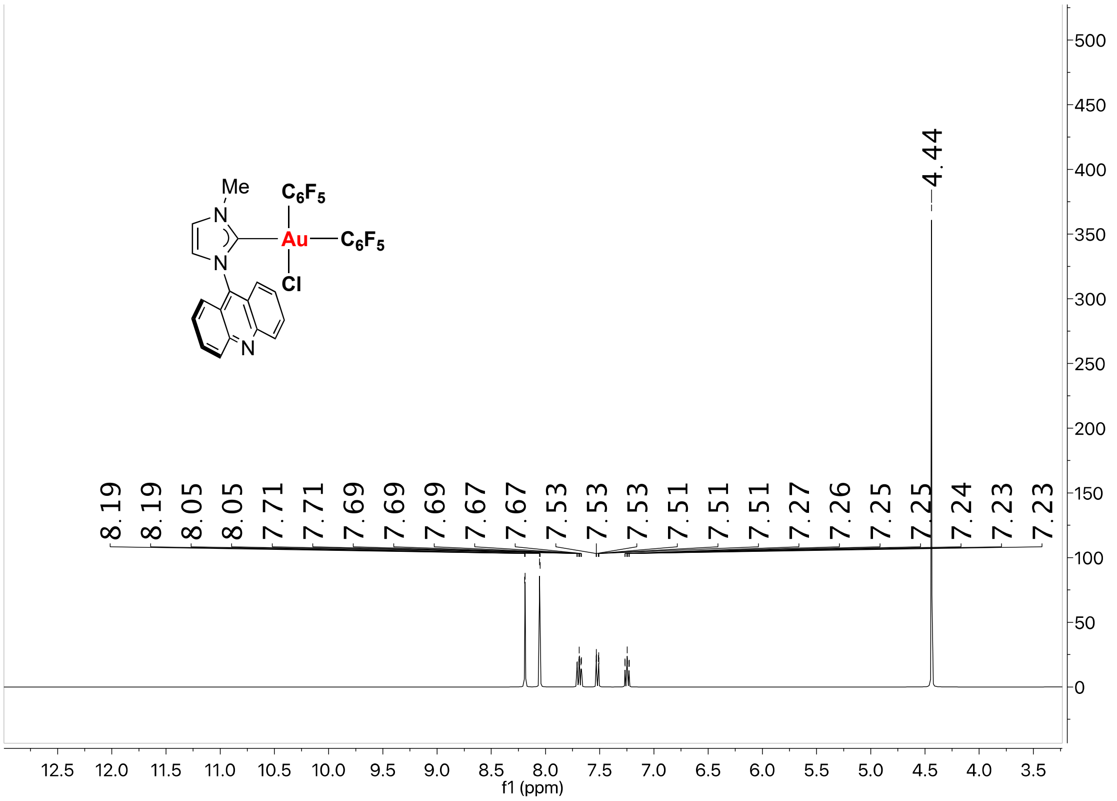

Figure S9. ^{1}H NMR spectrum of complex **2** in acetone-d^6.

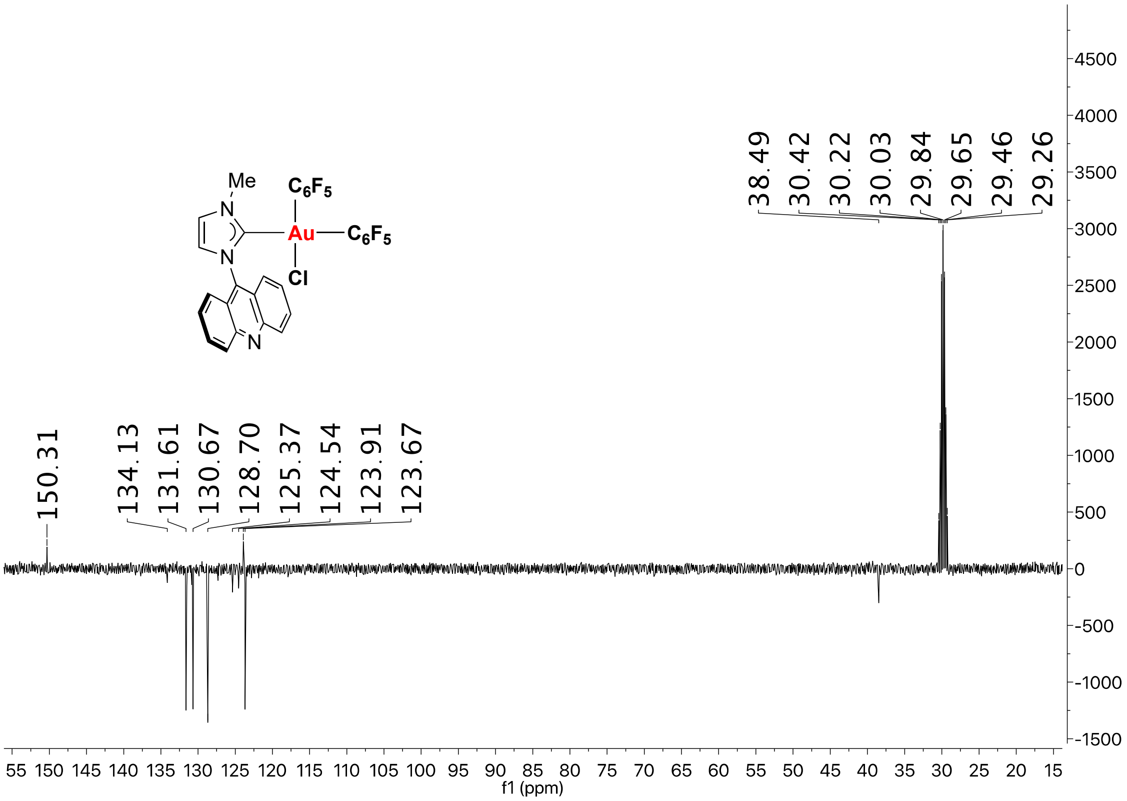

Figure S10. $^{13}C\text{-}\{^{1}H\}$ (APT) NMR spectrum of complex **2** in acetone-d6.

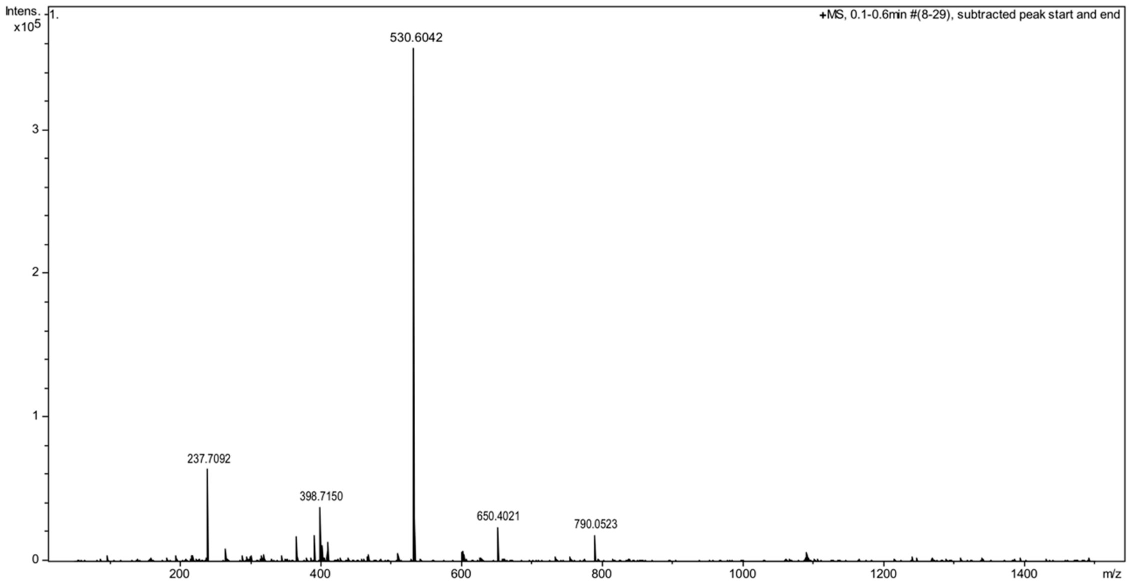

Figure S11. ESI+-MS spectrum of complex **2**.

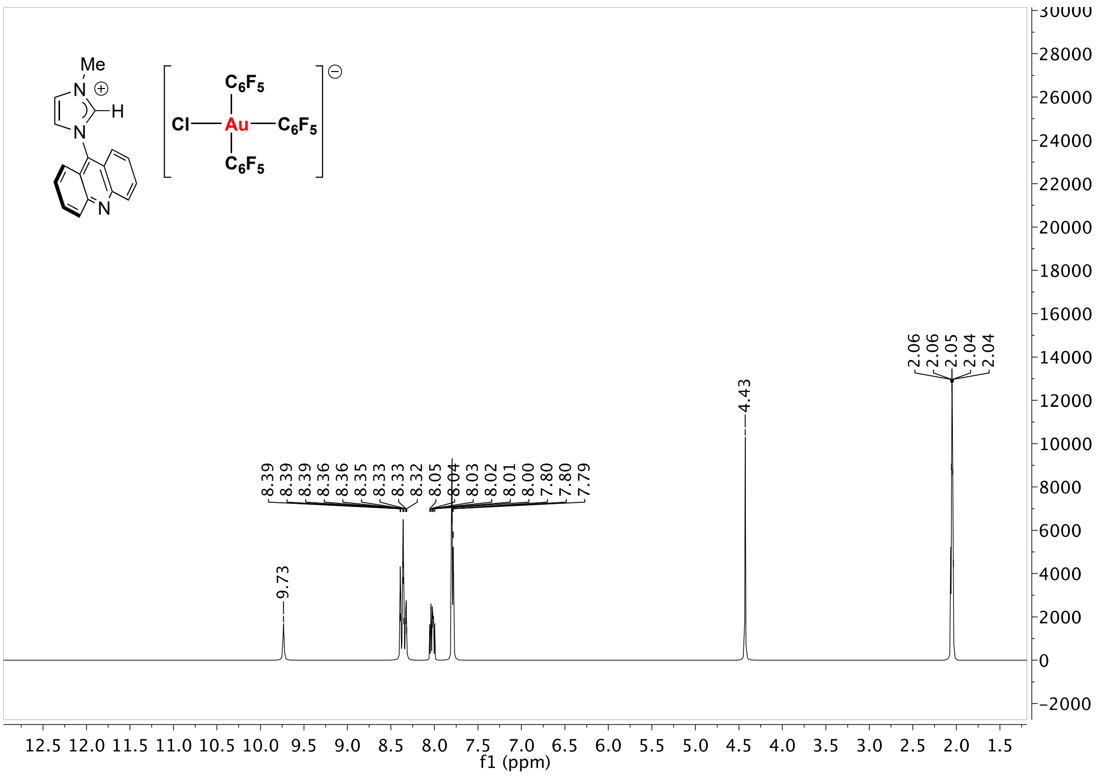

Figure S12. ^{1}H NMR spectrum of intermediate **3** in acetone-d^{6}.

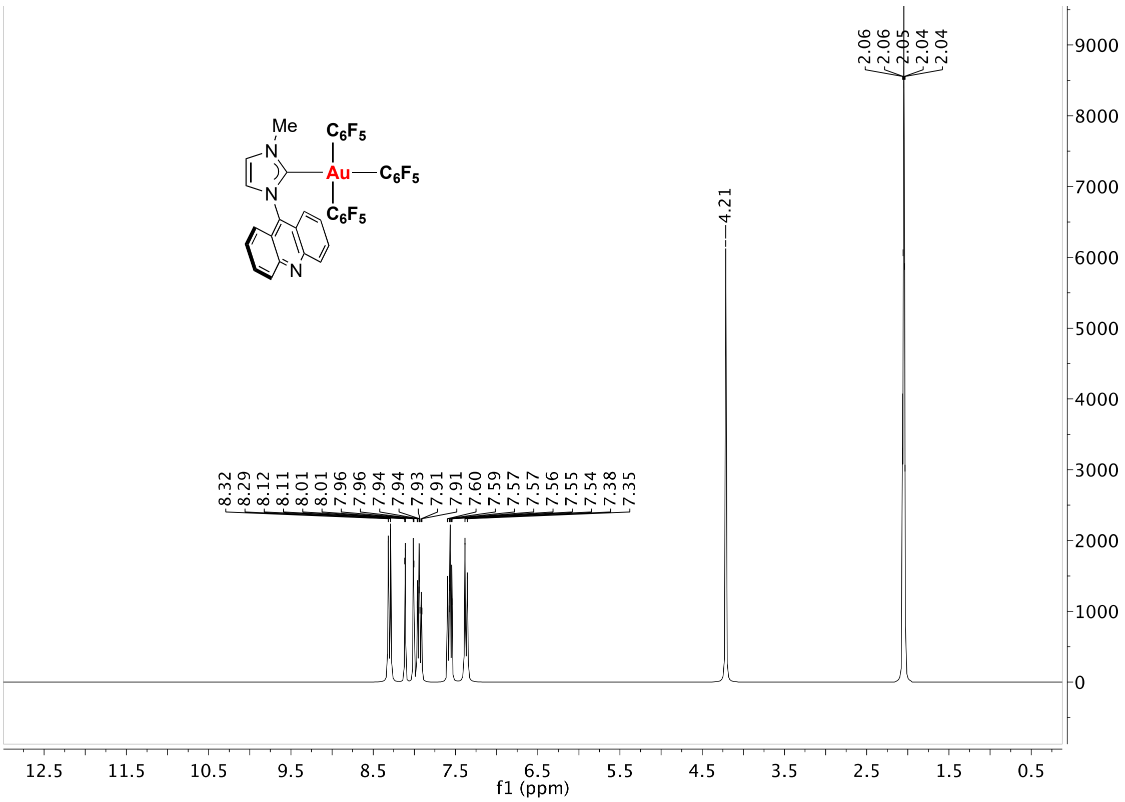

Figure S13. ^{1}H NMR spectrum of complex **3** in acetone-d^{6}.

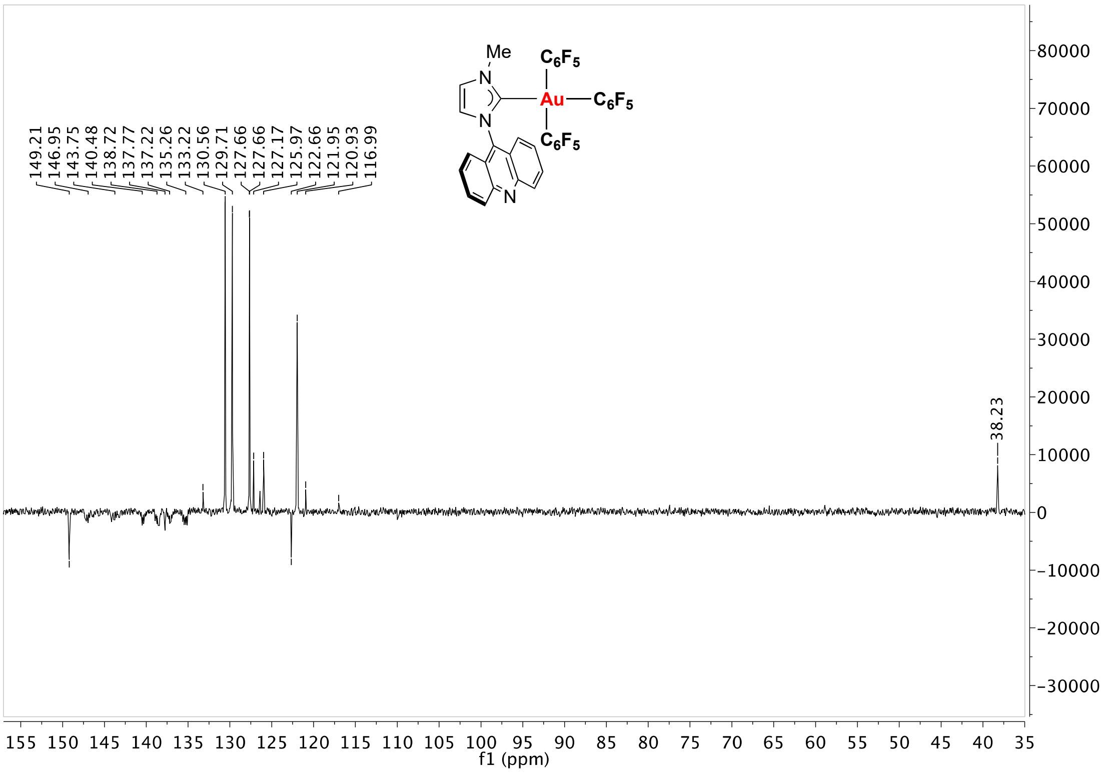

Figure S14. ^{13}C-{^{1}H} (APT) NMR spectrum of complex **3** in acetone-d^{6}.

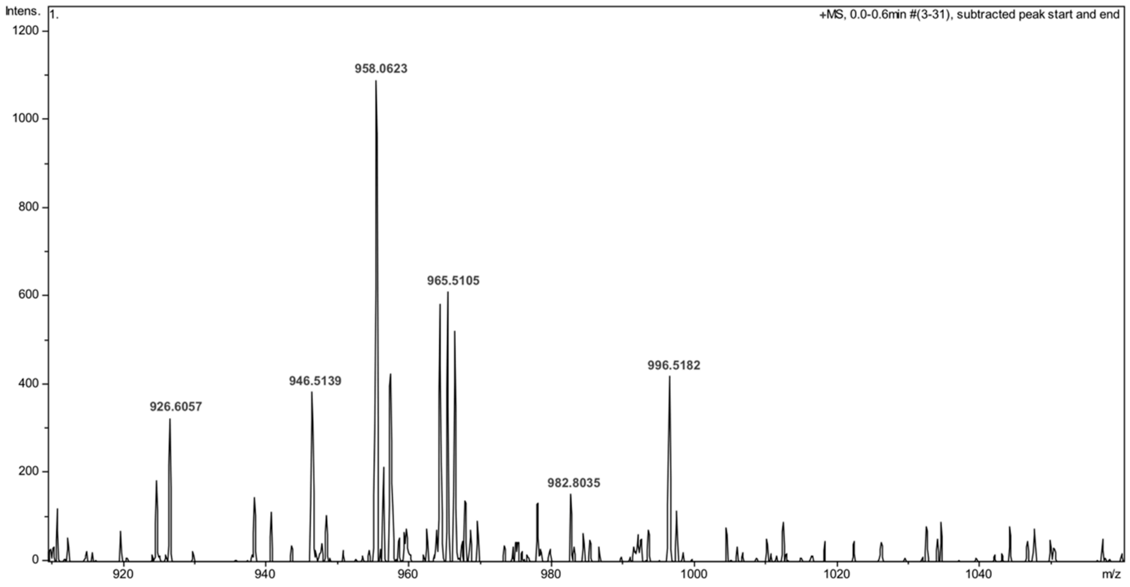

Figure S15. ESI^{+}-MS spectrum of complex **3**.

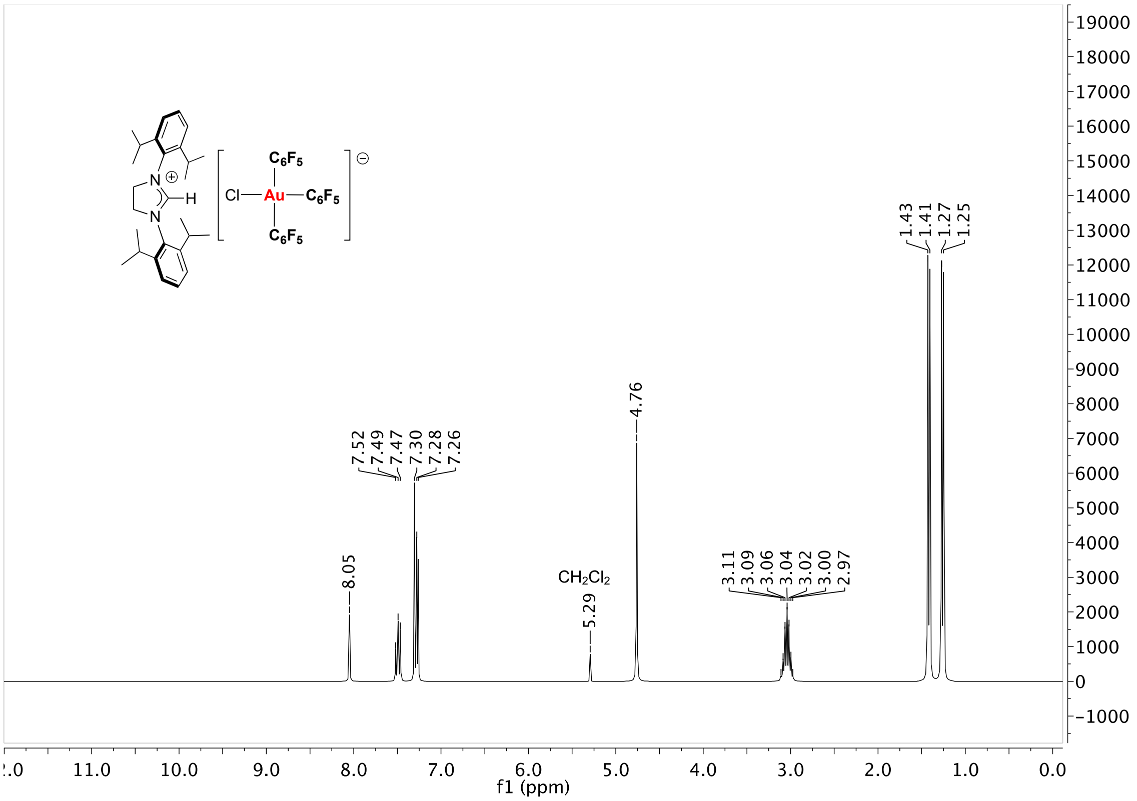

Figure S16. ^{1}H NMR spectrum of intermediate **4** in chloroform-d.

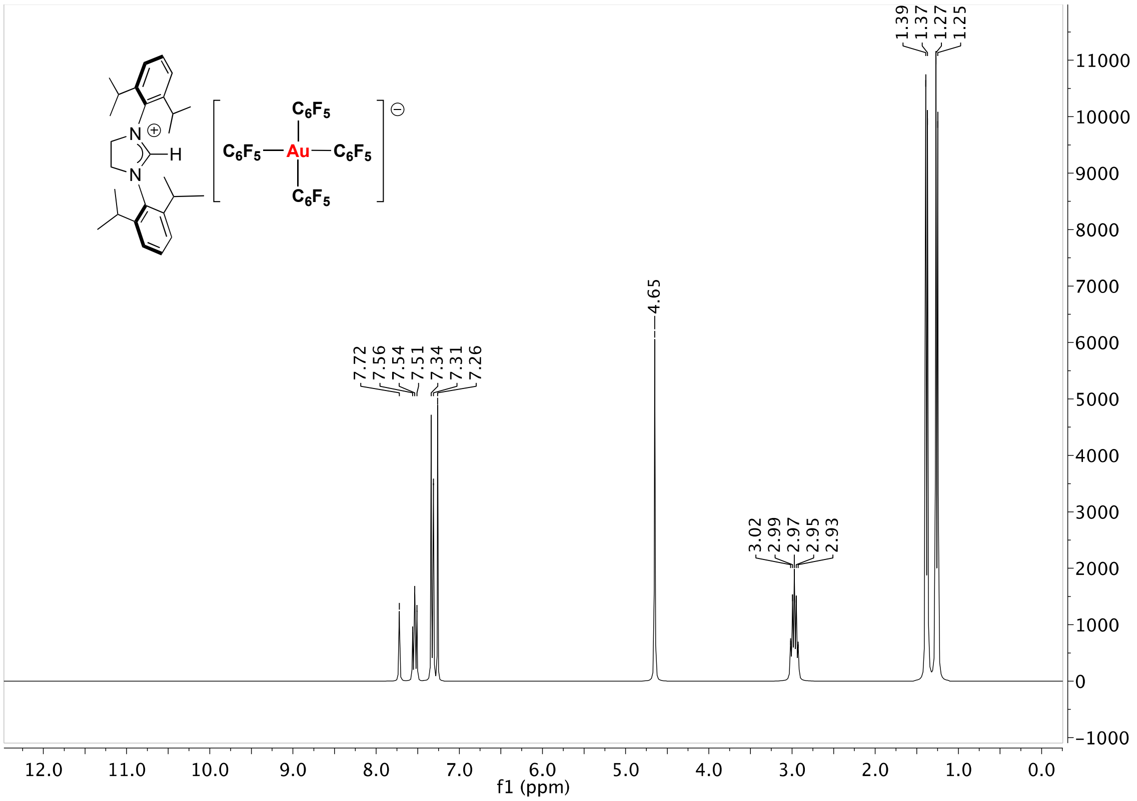

Figure S17. ^{1}H NMR spectrum of complex **4** in chloroform-d.

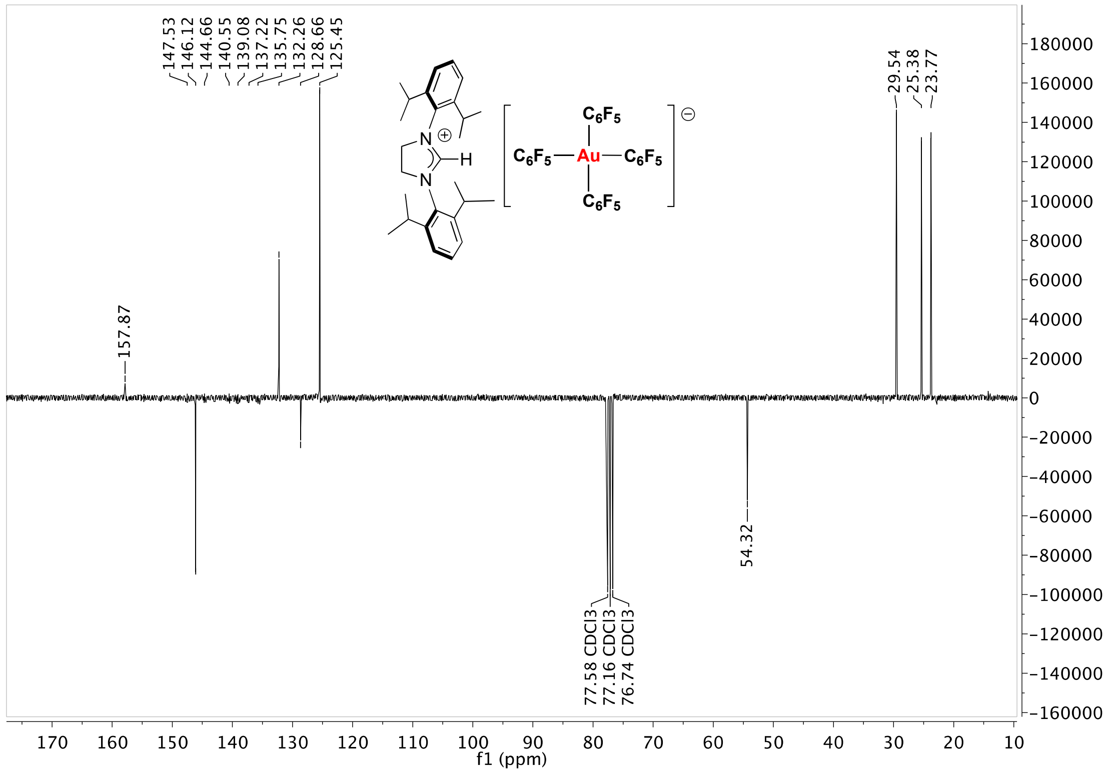

Figure S18. ^{13}C-{^{1}H} (APT) NMR spectrum of complex **4** in chloroform-d.

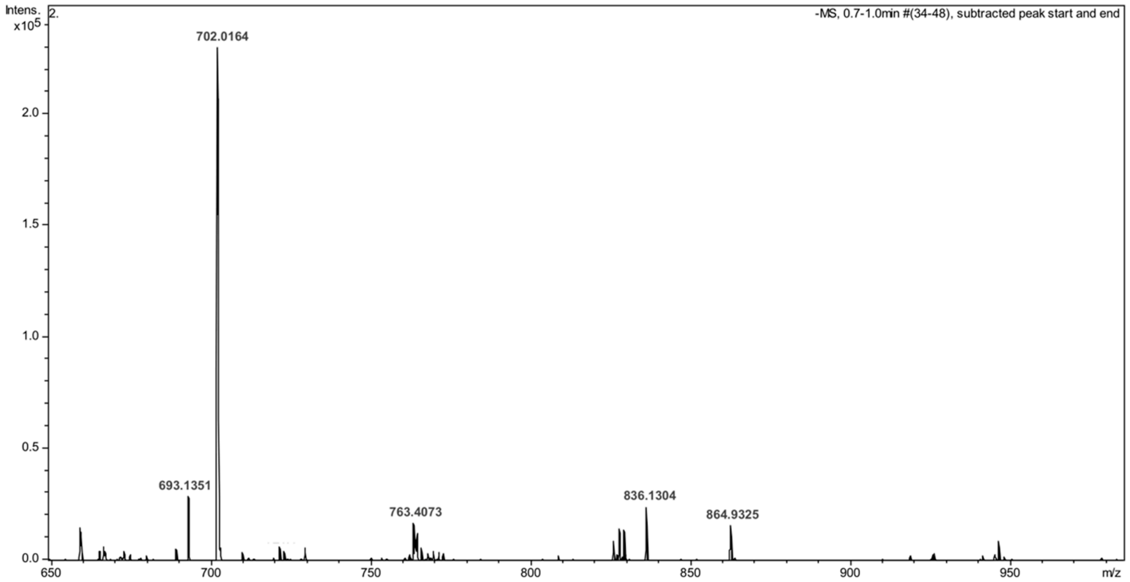

Figure S19. ESI^{-}-MS spectrum of complex **4**.

Crystallographic data. Crystal Structure Determinations. Crystals were mounted in inert oil on glass fibers and transferred to the cold gas stream of an Xcalibur Oxford Diffraction diffractometer or Bruker Apex Duo equipped with low-temperature attachments. Data were collected using monochromated Mo Kα radiation (λ = 0.71073 Å). The scan type was ω. Absorption corrections based on multiple scans were applied with the program SADABS,[51] or using spherical harmonics implemented in SCALE3 ABSPACK scaling algorithm.[52] The structures were solved with the ShelXS structure solution program using direct methods and by using Olex2 as the graphical interface.[53]

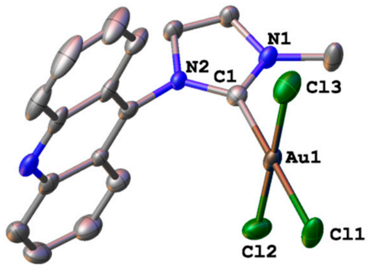

Figure S20. Solid structure of complex **1**.

Table S1. X-ray Crystallographic data for complex **1**.

Empirical formula	$C_{34}H_{26}Au_2Cl_6N_6$•$1.5CH_2Cl_2$
Formula weight	1252.63
Temperature/K	100.15
Crystal system	monoclinic
Space group	$P2_1/n$
a/Å	17.3553(3)
b/Å	13.9593(2)
c/Å	17.7709(3)
α/°	90
β/°	109.163(2)
γ/°	90
Volume/Å^3	4066.75(12)
Z	4
ρ_{calc}g/cm^3	2.046
μ/mm^{-1}	7.834
F(000)	2380.0
Crystal size/mm^3	0.42 × 0.26 × 0.2
Radiation	MoKα (λ = 0.71073)
2Θ range for data collection/°	8.434 to 50.994
Index ranges	-21 ≤ h ≤ 21, -16 ≤ k ≤ 16, -21 ≤ l ≤ 21
Reflections collected	39126
Independent reflections	7529 [R_{int} = 0.0352, R_{sigma} = 0.0250]
Data/restraints/parameters	7529/62/484
Goodness-of-fit on F^2	1.038
Final R indexes [I>=2σ (I)]	R_1 = 0.0368, wR_2 = 0.0816
Final R indexes [all data]	R_1 = 0.0423, wR_2 = 0.0845
Largest diff. peak/hole / e Å^{-3}	2.31/-1.43

Table S2. Selected bond lengths (Å) for complex **1**.

Atom	Atom	Length/Å	Atom	Atom	Length/Å
Au1	Cl1	2.3198(15)	N5	C20	1.378(9)
Au1	Cl2	2.2710(18)	N5	C22	1.457(9)
Au1	Cl3	2.2738(18)	N6	C28	1.266(9)
Au1	C1	2.007(6)	N6	C29	1.343(10)
N1	C1	1.333(7)	C18	Au3	1.911(7)
N1	C2	1.388(7)	C19	C20	1.342(10)
N1	C4	1.460(8)	C22	C23	1.489(8)
N2	C1	1.339(7)	C22	C34	1.386(9)
N2	C3	1.393(7)	C23	C24A	1.3900
N2	C5	1.446(7)	C23	C28	1.3900
N3	C11	1.333(9)	C23	C24B	1.403(5)
N3	C12	1.350(8)	C24A	C25A	1.3900
C2	C3	1.345(8)	C25A	C26A	1.3900
C5	C6	1.384(8)	C26A	C27A	1.3900
C5	C17	1.394(8)	C27A	C28	1.3900
C6	C7	1.424(9)	C28	C27B	1.413(5)
C6	C11	1.436(9)	C24B	C25B	1.390(5)
C7	C8	1.359(10)	C25B	C26B	1.390(5)
C8	C9	1.408(13)	C26B	C27B	1.393(5)
C9	C10	1.340(12)	C29	C30	1.417(11)
C10	C11	1.438(9)	C29	C34	1.433(9)
C12	C13	1.421(9)	C30	C31	1.339(13)
C12	C17	1.429(8)	C31	C32	1.415(11)
C13	C14	1.361(11)	C32	C33	1.351(10)
C14	C15	1.408(11)	C33	C34	1.425(9)
C15	C16	1.358(9)	Cl7	C35	1.712(13)
C16	C17	1.427(8)	Cl8	C35	1.800(13)
Au2	Cl4	2.319(6)	Cl9A	C36	1.883(9)
Au2	Cl5	2.213(3)	Cl10	Cl10[1]	1.601(19)
Au2	Cl6	2.295(6)	Cl10	C36	1.669(9)
Au2	C18	2.107(7)	Cl9B	C36	1.675(9)
Cl5	Au3	2.371(3)	Cl9	Cl9[1]	1.05(2)
N4	C18	1.318(10)	Cl9	C36	1.897(9)
N4	C19	1.374(9)	Au3	Cl11	2.327(6)

[1]1-X,1-Y,1-Z

Table S3. Selected bond angles (°) for complex **1**.

Atom	Atom	Atom	Angle/°	Atom	Atom	Atom	Angle/°
Cl2	Au1	Cl1	91.41(7)	C20	N5	C22	123.7(5)
Cl2	Au1	Cl3	177.62(6)	C28	N6	C29	118.7(6)
Cl3	Au1	Cl1	90.93(7)	N4	C18	Au2	131.1(5)
C1	Au1	Cl1	178.74(17)	N4	C18	N5	106.0(6)
C1	Au1	Cl2	89.52(17)	N4	C18	Au3	121.1(5)
C1	Au1	Cl3	88.15(17)	N5	C18	Au2	122.8(5)
C1	N1	C2	110.0(5)	N5	C18	Au3	132.8(6)
C1	N1	C4	125.7(5)	C20	C19	N4	107.5(7)
C2	N1	C4	124.3(5)	C19	C20	N5	105.8(6)
C1	N2	C3	109.6(5)	N5	C22	C23	121.7(5)

C1	N2	C5	126.4(5)	C34	C22	N5	118.3(6)
C3	N2	C5	124.0(5)	C34	C22	C23	119.9(6)
C11	N3	C12	119.1(5)	C24A	C23	C22	126.9(4)
N1	C1	Au1	125.7(4)	C24A	C23	C28	120.0
N1	C1	N2	106.9(5)	C28	C23	C22	112.9(4)
N2	C1	Au1	127.2(4)	C28	C23	C24B	136.1(6)
C3	C2	N1	106.8(5)	C24B	C23	C22	110.9(6)
C2	C3	N2	106.7(5)	C23	C24A	C25A	120.0
C6	C5	N2	118.4(5)	C26A	C25A	C24A	120.0
C6	C5	C17	122.4(6)	C25A	C26A	C27A	120.0
C17	C5	N2	119.1(5)	C28	C27A	C26A	120.0
C5	C6	C7	124.8(6)	N6	C28	C23	128.8(5)
C5	C6	C11	116.0(6)	N6	C28	C27A	111.2(5)
C7	C6	C11	119.3(6)	N6	C28	C27B	135.4(7)
C8	C7	C6	120.1(7)	C23	C28	C27B	95.7(6)
C7	C8	C9	120.6(8)	C27A	C28	C23	120.0
C10	C9	C8	121.8(7)	C25B	C24B	C23	120.7(11)
C9	C10	C11	120.3(7)	C24B	C25B	C26B	110.7(12)
N3	C11	C6	123.4(6)	C25B	C26B	C27B	120.0(13)
N3	C11	C10	118.6(6)	C26B	C27B	C28	136.6(12)
C6	C11	C10	118.0(7)	N6	C29	C30	118.3(7)
N3	C12	C13	119.0(6)	N6	C29	C34	122.5(7)
N3	C12	C17	122.3(6)	C30	C29	C34	119.2(7)
C13	C12	C17	118.7(6)	C31	C30	C29	120.4(7)
C14	C13	C12	120.3(7)	C30	C31	C32	121.1(8)
C13	C14	C15	120.8(7)	C33	C32	C31	120.8(8)
C16	C15	C14	121.1(7)	C32	C33	C34	120.4(6)
C15	C16	C17	119.8(6)	C22	C34	C29	117.2(6)
C5	C17	C12	116.7(6)	C22	C34	C33	124.7(6)
C5	C17	C16	124.1(5)	C33	C34	C29	118.1(6)
C16	C17	C12	119.2(6)	Cl7	C35	Cl8	112.5(7)
Cl5	Au2	Cl4	91.01(15)	Cl10[1]	Cl10	C36	133.4(12)
Cl5	Au2	Cl6	174.84(14)	Cl9[1]	Cl9	C36	133.9(15)
Cl6	Au2	Cl4	90.62(19)	Cl10	C36	Cl9A	110.6(6)
C18	Au2	Cl4	175.8(3)	Cl9B	C36	Cl9	103.1(6)
C18	Au2	Cl5	86.75(19)	C18	Au3	Cl5	87.0(2)
C18	Au2	Cl6	91.3(2)	C18	Au3	Cl11	173.0(3)
C18	N4	C19	110.4(6)	C18	Au3	Cl12	89.5(2)

[1] 1-X,1-Y,1-Z

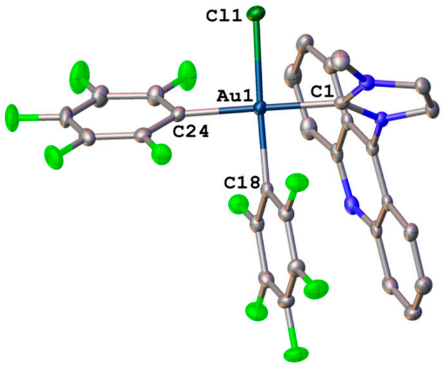

Figure S21. Solid structure of complex **2**.

Table S4. X-ray Crystallographic data for complex **2**.

Empirical formula	$C_{32}AuClF_{10}N_3H_{19}O$
Formula weight	883.92
Temperature/K	100.15
Crystal system	triclinic
Space group	P-1
a/Å	11.7486(2)
b/Å	11.8230(2)
c/Å	12.0308(2)
α/°	73.6670(10)
β/°	82.4210(10)
γ/°	70.058(2)
Volume/Å^3	1506.32(5)
Z	2
ρ_{calc}g/cm^3	1.949
μ/mm^{-1}	5.067
F(000)	852.0
Crystal size/mm^3	0.18 × 0.12 × 0.1
Radiation	MoKα (λ = 0.71073)
2Θ range for data collection/°	8.314 to 51
Index ranges	-14 ≤ h ≤ 14, -14 ≤ k ≤ 14, -14 ≤ l ≤ 14
Reflections collected	28980
Independent reflections	5594 [R_{int} = 0.0274, R_{sigma} = 0.0165]
Data/restraints/parameters	5594/0/433
Goodness-of-fit on F^2	1.073
Final R indexes [I>=2σ (I)]	R_1 = 0.0146, wR_2 = 0.0370
Final R indexes [all data]	R_1 = 0.0152, wR_2 = 0.0373
Largest diff. peak/hole / e Å^{-3}	0.79/-0.50

Table S5. Selected bond lengths (Å) for complex **2**.

Atom	Atom	Length/Å	Atom	Atom	Length/Å
Au(1)	Cl(1)	2.3323(6)	C(6)	C(11)	1.438(3)
Au(1)	C(1)	2.047(2)	C(7)	C(8)	1.361(3)
Au(1)	C(18)	2.036(2)	C(8)	C(9)	1.421(4)

Au(1)	C(24)	2.053(2)	C(9)	C(10)	1.360(4)
F(1)	C(19)	1.351(3)	C(10)	C(11)	1.427(3)
F(2)	C(20)	1.342(3)	C(12)	C(13)	1.431(3)
F(3)	C(21)	1.342(3)	C(12)	C(17)	1.437(3)
F(4)	C(22)	1.343(3)	C(13)	C(14)	1.357(4)
F(5)	C(23)	1.346(2)	C(14)	C(15)	1.415(4)
F(6)	C(25)	1.353(3)	C(15)	C(16)	1.361(3)
F(7)	C(26)	1.345(3)	C(16)	C(17)	1.428(3)
F(8)	C(27)	1.347(3)	C(18)	C(19)	1.383(3)
F(9)	C(28)	1.345(3)	C(18)	C(23)	1.372(3)
F(10)	C(29)	1.350(3)	C(19)	C(20)	1.378(3)
N(1)	C(1)	1.333(3)	C(20)	C(21)	1.378(3)
N(1)	C(2)	1.381(3)	C(21)	C(22)	1.375(3)
N(1)	C(4)	1.464(3)	C(22)	C(23)	1.384(3)
N(2)	C(1)	1.353(3)	C(24)	C(25)	1.377(3)
N(2)	C(3)	1.394(3)	C(24)	C(29)	1.383(3)
N(2)	C(5)	1.435(3)	C(25)	C(26)	1.382(3)
N(3)	C(11)	1.347(3)	C(26)	C(27)	1.373(4)
N(3)	C(12)	1.337(3)	C(27)	C(28)	1.373(4)
C(2)	C(3)	1.342(3)	C(28)	C(29)	1.376(3)
C(5)	C(6)	1.403(3)	O(1)	C(30)	1.204(3)
C(5)	C(17)	1.396(3)	C(30)	C(31)	1.482(4)
C(6)	C(7)	1.422(3)	C(30)	C(32)	1.482(4)

Table S6. Selected bond angles (°) for complex **2**.

Atom	Atom	Atom	Angle/°	Atom	Atom	Atom	Angle/°
C(1)	Au(1)	Cl(1)	89.18(6)	C(16)	C(17)	C(12)	118.9(2)
C(1)	Au(1)	C(24)	179.03(8)	C(19)	C(18)	Au(1)	119.86(16)
C(18)	Au(1)	Cl(1)	176.82(6)	C(23)	C(18)	Au(1)	122.87(16)
C(18)	Au(1)	C(1)	88.27(8)	C(23)	C(18)	C(19)	117.1(2)
C(18)	Au(1)	C(24)	91.06(9)	F(1)	C(19)	C(18)	120.5(2)
C(24)	Au(1)	Cl(1)	91.51(6)	F(1)	C(19)	C(20)	117.11(19)
C(1)	N(1)	C(2)	110.45(19)	C(20)	C(19)	C(18)	122.4(2)
C(1)	N(1)	C(4)	125.48(19)	F(2)	C(20)	C(19)	120.9(2)
C(2)	N(1)	C(4)	124.07(19)	F(2)	C(20)	C(21)	119.9(2)
C(1)	N(2)	C(3)	109.88(18)	C(19)	C(20)	C(21)	119.2(2)
C(1)	N(2)	C(5)	124.53(18)	F(3)	C(21)	C(20)	120.2(2)
C(3)	N(2)	C(5)	125.35(19)	F(3)	C(21)	C(22)	120.1(2)
C(12)	N(3)	C(11)	118.53(19)	C(22)	C(21)	C(20)	119.7(2)
N(1)	C(1)	Au(1)	127.78(17)	F(4)	C(22)	C(21)	119.4(2)
N(1)	C(1)	N(2)	106.01(19)	F(4)	C(22)	C(23)	120.8(2)
N(2)	C(1)	Au(1)	126.21(16)	C(21)	C(22)	C(23)	119.8(2)
C(3)	C(2)	N(1)	107.4(2)	F(5)	C(23)	C(18)	120.7(2)
C(2)	C(3)	N(2)	106.2(2)	F(5)	C(23)	C(22)	117.5(2)

C(6)	C(5)	N(2)	118.50(19)	C(18)	C(23)	C(22)	121.8(2)
C(17)	C(5)	N(2)	120.04(19)	C(25)	C(24)	Au(1)	122.47(17)
C(17)	C(5)	C(6)	121.4(2)	C(25)	C(24)	C(29)	115.8(2)
C(5)	C(6)	C(7)	124.5(2)	C(29)	C(24)	Au(1)	121.72(17)
C(5)	C(6)	C(11)	116.56(19)	F(6)	C(25)	C(24)	119.7(2)
C(7)	C(6)	C(11)	119.0(2)	F(6)	C(25)	C(26)	117.2(2)
C(8)	C(7)	C(6)	120.3(2)	C(24)	C(25)	C(26)	123.1(2)
C(7)	C(8)	C(9)	120.9(2)	F(7)	C(26)	C(25)	120.9(2)
C(10)	C(9)	C(8)	120.5(2)	F(7)	C(26)	C(27)	119.9(2)
C(9)	C(10)	C(11)	120.6(2)	C(27)	C(26)	C(25)	119.2(2)
N(3)	C(11)	C(6)	123.2(2)	F(8)	C(27)	C(26)	120.3(2)
N(3)	C(11)	C(10)	118.1(2)	F(8)	C(27)	C(28)	120.1(2)
C(10)	C(11)	C(6)	118.7(2)	C(28)	C(27)	C(26)	119.6(2)
N(3)	C(12)	C(13)	117.9(2)	F(9)	C(28)	C(27)	119.4(2)
N(3)	C(12)	C(17)	123.5(2)	F(9)	C(28)	C(29)	120.8(2)
C(13)	C(12)	C(17)	118.5(2)	C(27)	C(28)	C(29)	119.7(2)
C(14)	C(13)	C(12)	120.4(2)	F(10)	C(29)	C(24)	120.1(2)
C(13)	C(14)	C(15)	121.0(2)	F(10)	C(29)	C(28)	117.2(2)
C(16)	C(15)	C(14)	120.8(2)	C(28)	C(29)	C(24)	122.6(2)
C(15)	C(16)	C(17)	120.2(2)	O(1)	C(30)	C(31)	121.4(3)
C(5)	C(17)	C(12)	116.7(2)	O(1)	C(30)	C(32)	122.4(3)
C(5)	C(17)	C(16)	124.4(2)	C(31)	C(30)	C(32)	116.2(3)

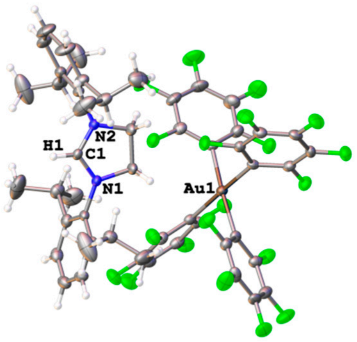

Figure S22. Solid structure of salt **4**.

Table S7. X-ray Crystallographic data for salt **4**.

Empirical formula	$C_{51}H_{39}N_2F_{20}Au$
Formula weight	1256.81
Temperature/K	100
Crystal system	monoclinic
Space group	$P2_1/c$
a/Å	18.350(4)
b/Å	10.560(2)
c/Å	25.490(5)
α/°	90
β/°	101.83(3)
γ/°	90
Volume/Å^3	4834.4(18)
Z	4

ρ_{calc}g/cm^3	1.727
μ/mm^{-1}	3.159
F(000)	2472.0
Crystal size/mm^3	0.24 × 0.16 × 0.06
Radiation	MoKα (λ = 0.71073)
2Θ range for data collection/°	3.264 to 51
Index ranges	-22 ≤ h ≤ 22, -12 ≤ k ≤ 12, -30 ≤ l ≤ 30
Reflections collected	37345
Independent reflections	9001 [R_{int} = 0.0503, R_{sigma} = 0.0415]
Data/restraints/parameters	9001/0/667
Goodness-of-fit on F^2	1.019
Final R indexes [I>=2σ (I)]	R_1 = 0.0256, wR_2 = 0.0587
Final R indexes [all data]	R_1 = 0.0323, wR_2 = 0.0615
Largest diff. peak/hole / e Å^{-3}	1.19/-0.73

Table S8. Selected bond lengths (Å) for salt **4**.

Atom	Atom	Length/Å	Atom	Atom	Length/Å
Au1	C28	2.044(3)	C10	C12	1.512(5)
Au1	C34	2.053(3)	C13	C14	1.515(5)
Au1	C40	2.059(3)	C13	C15	1.518(5)
Au1	C46	2.061(3)	C16	C17	1.387(5)
F1	C35	1.348(4)	C16	C21	1.403(5)
F2	C36	1.344(4)	C17	C18	1.393(5)
F3	C37	1.333(4)	C17	C22	1.516(5)
F4	C38	1.342(4)	C18	C19	1.367(5)
F5	C39	1.359(3)	C19	C20	1.374(6)
F6	C29	1.350(3)	C20	C21	1.388(5)
F7	C30	1.342(4)	C21	C25	1.512(5)
F8	C31	1.340(4)	C22	C23	1.531(6)
F9	C32	1.344(4)	C22	C24	1.518(5)
F10	C33	1.354(4)	C25	C26	1.491(6)
F11	C41	1.352(4)	C25	C27	1.525(6)
F12	C42	1.357(4)	C28	C29	1.382(4)
F13	C43	1.351(3)	C28	C33	1.375(4)
F14	C44	1.329(4)	C29	C30	1.370(5)
F15	C45	1.355(4)	C30	C31	1.381(5)
F16	C47	1.351(4)	C31	C32	1.370(5)
F17	C48	1.349(4)	C32	C33	1.367(5)
F18	C49	1.345(4)	C34	C35	1.373(4)
F19	C50	1.341(4)	C34	C39	1.377(5)
F20	C51	1.347(4)	C35	C36	1.373(5)
N1	C1	1.308(4)	C36	C37	1.371(5)
N1	C2	1.475(4)	C37	C38	1.374(5)
N1	C4	1.437(4)	C38	C39	1.373(5)
N2	C1	1.297(4)	C40	C41	1.370(4)
N2	C3	1.480(4)	C40	C45	1.375(4)
N2	C16	1.443(4)	C41	C42	1.375(4)
C2	C3	1.535(4)	C42	C43	1.360(5)
C4	C5	1.394(4)	C43	C44	1.374(5)

C4	C9	1.396(4)	C44	C45	1.374(4)
C5	C6	1.391(5)	C46	C47	1.374(4)
C5	C10	1.520(5)	C46	C51	1.373(5)
C6	C7	1.374(5)	C47	C48	1.378(5)
C7	C8	1.372(5)	C48	C49	1.361(5)
C8	C9	1.390(4)	C49	C50	1.366(5)
C9	C13	1.519(5)	C50	C51	1.382(5)
C10	C11	1.519(5)			

Table S6. Selected bond angles (°) for complex **2**.

Atom	Atom	Atom	Angle/°	Atom	Atom	Atom	Angle/°
C28	Au1	C34	176.43(12)	C32	C31	C30	119.5(3)
C28	Au1	C40	88.06(12)	F9	C32	C31	119.6(3)
C28	Au1	C46	91.90(12)	F9	C32	C33	121.5(3)
C34	Au1	C40	88.84(12)	C33	C32	C31	118.8(3)
C34	Au1	C46	91.33(12)	F10	C33	C28	119.1(3)
C40	Au1	C46	175.47(12)	F10	C33	C32	116.7(3)
C1	N1	C2	109.6(3)	C32	C33	C28	124.2(3)
C1	N1	C4	126.7(3)	C35	C34	Au1	121.8(2)
C4	N1	C2	123.0(2)	C35	C34	C39	115.3(3)
C1	N2	C3	109.9(2)	C39	C34	Au1	122.8(2)
C1	N2	C16	125.1(3)	F1	C35	C34	119.7(3)
C16	N2	C3	125.0(3)	F1	C35	C36	117.0(3)
N2	C1	N1	114.7(3)	C36	C35	C34	123.2(3)
N1	C2	C3	103.1(2)	F2	C36	C35	120.3(3)
N2	C3	C2	102.7(3)	F2	C36	C37	120.0(3)
C5	C4	N1	118.7(3)	C37	C36	C35	119.7(3)
C5	C4	C9	123.0(3)	F3	C37	C36	121.0(3)
C9	C4	N1	118.3(3)	F3	C37	C38	120.1(3)
C4	C5	C10	123.2(3)	C36	C37	C38	119.0(3)
C6	C5	C4	117.1(3)	F4	C38	C37	119.3(3)
C6	C5	C10	119.7(3)	F4	C38	C39	121.1(3)
C7	C6	C5	121.2(3)	C39	C38	C37	119.6(3)
C8	C7	C6	120.5(3)	F5	C39	C34	119.5(3)
C7	C8	C9	121.2(3)	F5	C39	C38	117.3(3)
C4	C9	C13	123.0(3)	C38	C39	C34	123.2(3)
C8	C9	C4	117.0(3)	C41	C40	Au1	121.0(2)
C8	C9	C13	119.9(3)	C41	C40	C45	115.7(3)
C11	C10	C5	112.5(3)	C45	C40	Au1	123.2(2)
C12	C10	C5	110.0(3)	F11	C41	C40	120.1(3)
C12	C10	C11	110.0(3)	F11	C41	C42	117.1(3)
C14	C13	C9	110.4(3)	C40	C41	C42	122.7(3)
C14	C13	C15	110.7(4)	F12	C42	C41	121.3(3)
C15	C13	C9	111.0(3)	F12	C42	C43	119.2(3)
C17	C16	N2	118.7(3)	C43	C42	C41	119.5(3)
C17	C16	C21	123.2(3)	F13	C43	C42	119.3(3)
C21	C16	N2	118.1(3)	F13	C43	C44	120.5(3)
C16	C17	C18	116.9(3)	C42	C43	C44	120.2(3)
C16	C17	C22	122.8(3)	F14	C44	C43	119.9(3)

C18	C17	C22	120.2(3)	F14	C44	C45	121.6(3)
C19	C18	C17	121.3(4)	C45	C44	C43	118.4(3)
C18	C19	C20	120.6(3)	F15	C45	C40	119.5(3)
C19	C20	C21	121.2(3)	F15	C45	C44	117.1(3)
C16	C21	C25	122.7(3)	C44	C45	C40	123.4(3)
C20	C21	C16	116.7(3)	C47	C46	Au1	124.9(2)
C20	C21	C25	120.5(3)	C51	C46	Au1	119.8(2)
C17	C22	C23	110.6(3)	C51	C46	C47	115.2(3)
C17	C22	C24	112.3(3)	F16	C47	C46	119.9(3)
C24	C22	C23	110.4(4)	F16	C47	C48	117.2(3)
C21	C25	C27	111.0(3)	C46	C47	C48	122.9(3)
C26	C25	C21	114.5(4)	F17	C48	C47	120.4(3)
C26	C25	C27	108.2(3)	F17	C48	C49	119.7(3)
C29	C28	Au1	122.2(2)	C49	C48	C47	119.9(3)
C33	C28	Au1	122.6(2)	F18	C49	C48	120.2(3)
C33	C28	C29	114.9(3)	F18	C49	C50	120.3(3)
F6	C29	C28	119.7(3)	C48	C49	C50	119.5(3)
F6	C29	C30	117.2(3)	F19	C50	C49	120.0(3)
C30	C29	C28	123.1(3)	F19	C50	C51	121.0(3)
F7	C30	C29	121.5(3)	C49	C50	C51	119.1(3)
F7	C30	C31	119.1(3)	F20	C51	C46	119.8(3)
C29	C30	C31	119.4(3)	F20	C51	C50	116.8(3)
F8	C31	C30	119.6(3)	C46	C51	C50	123.4(3)
F8	C31	C32	120.8(3)				

Computational details

In order to estimate the formation energies of the different NHC-Au(III) complexes, some computational calculations have been carried out on the NHC and Au(III) fragments, as well as on the arising complex, on the framework of the DFT methods. Using the crystallographic structures as starting point (in those species that have been no reported crystal was used a starting crystal structure of a comparable species), geometry optimization calculations have been performed at the PBEh-3c level. As reported in the literature, this kind of functionals have been used in the estimation of relative energies and bonding interactions of NHC-Au(III) complexes producing reliable results.[54–56] In all calculations, the all electron Ahlrichs def2-SVP basis set has been used for all atoms, except for Au and Cl where have been used the def2-TZVP and def2-TZVP(-f) versions, respectively.[57,58] The geometrical counterpoise correction, to treat the basis set superposition errors (BSSE),[59] and the Grimme correction,[60] to include the London dispersion effects, were accounted for by the gCP and D3 schemes, respectively, as implemented in the ORCA 4.2.1 package.[61,62] The resolution of identity approach has also been considered by using the def2/J auxiliary basis set for Coulomb integrals computation. The solvent effect was also added by the Conductor-like Polarizable Continuum Model (CPCM).[63] In the simulations we have used dichloromethane as solvent (dielectric constant = 9.08 and refractive index = 1.424). The threshold for the energy convergence in the self-consistent field procedure was $1x10^{-8}$ a.u. No negative normal modes were obtained by analytical frequency calculations on the optimized geometries. Time Dependent DFT calculations have been also carried out to simulate the absorption spectrum of complex **3**. 50 excited singlet states have been calculated. To analize the transitions, localized orbitals, according to the Pipek-Mezey (population-localization) scheme, were computed by default with the orca_cis module within the ORCA package. A Gaussian fitting with a linewidth of 50 nm was used for plotting the spectrum curves. The Avogadro visualization tools[64] have been used for plotting geometries and orbitals (isovalue 0.05).

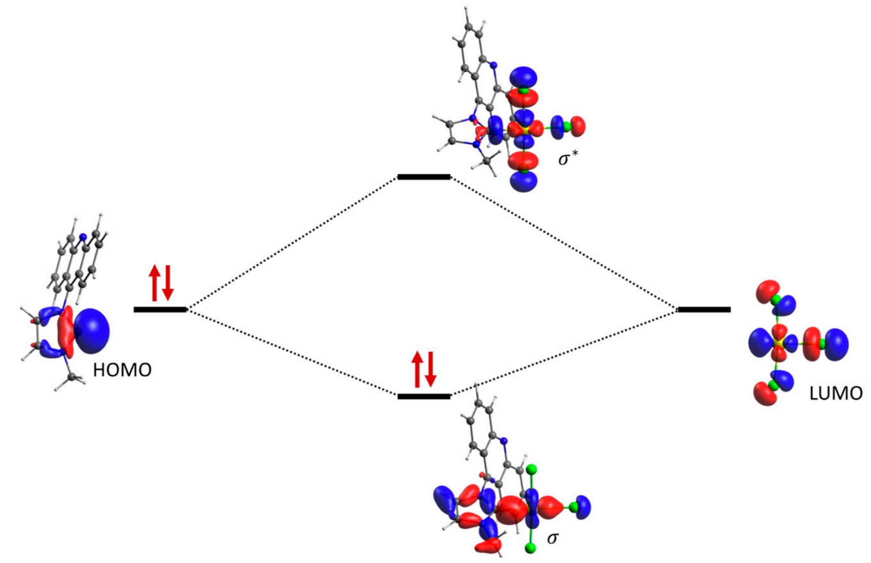

Figure S23. Diagram for the σ interaction between 1-(9-acridine)-3-methylimidazol-2-ylidene and [$AuCl_3$] species enabling the formation of complex **1**. b) Lowest energy particle, or LUMO, of complex **1**.

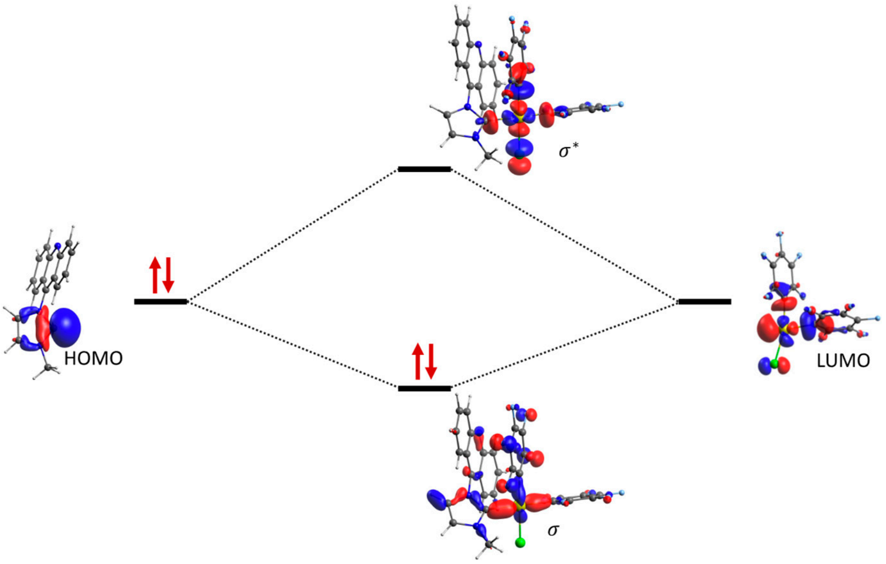

Figure S24. Diagram for the σ interaction between 1-(9-acridine)-3-methylimidazolylidene and [$AuCl(C_6F_5)_2$] species enabling the formation of complex **2**.

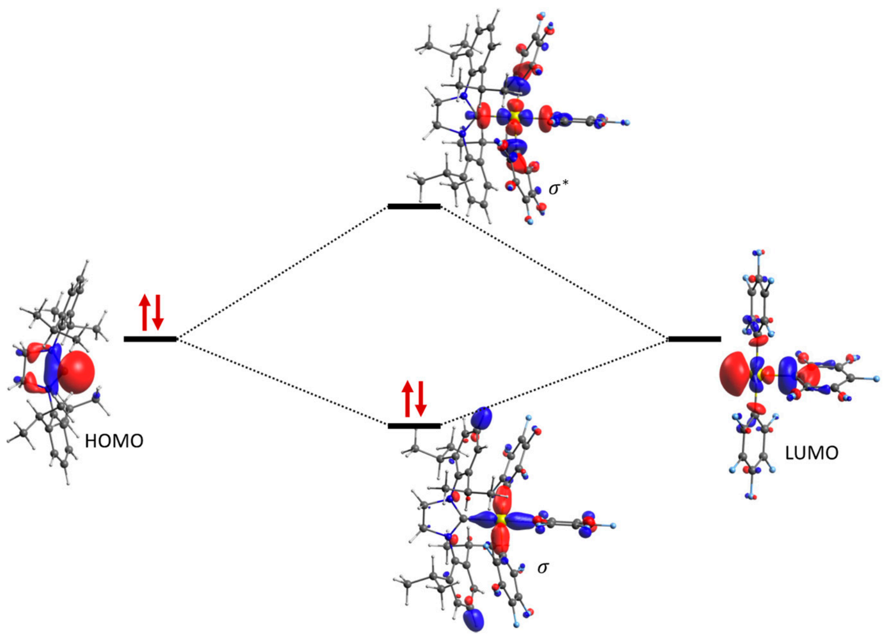

Figure S25. Diagram for the σ interaction between 1,3-Bis(2,6-diisopropylphenyl)imidazolidin-2-ylidene (SIPr) and $[Au(C_6F_5)_3]$ species enabling the formation of complex **4***.

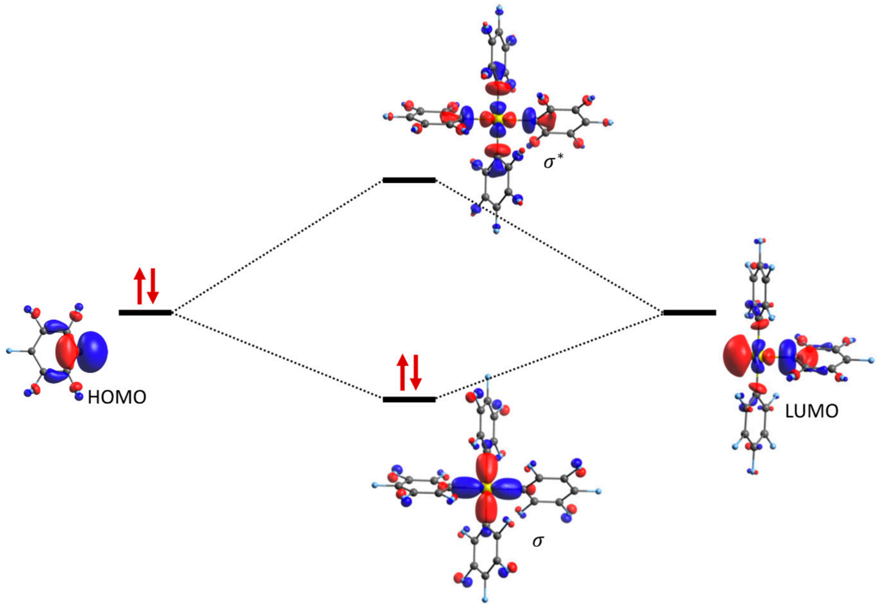

Figure S26. Diagram for the σ interaction between $[C_6F_5]^-$ fragment and $[Au(C_6F_3)_3]$ species enabling the formation of anion **4**.

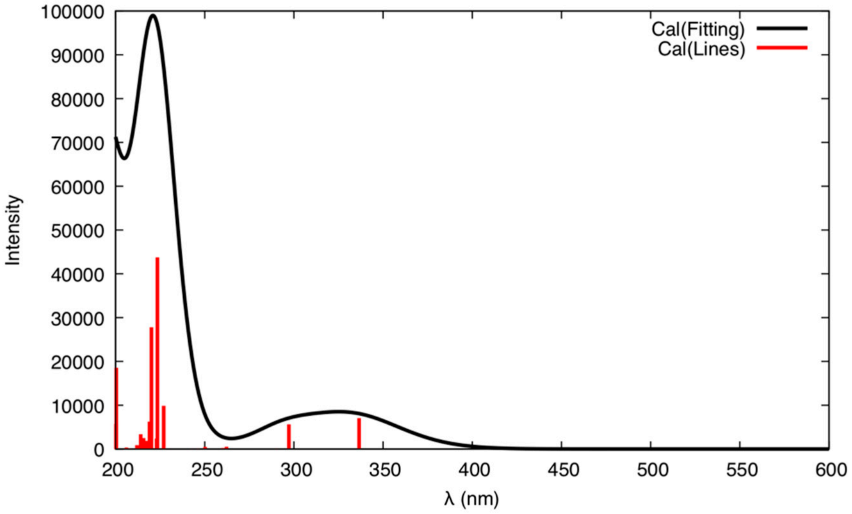

Figure S27. Calculated absorption spectrum via transition electric dipole moments of complex **3**.

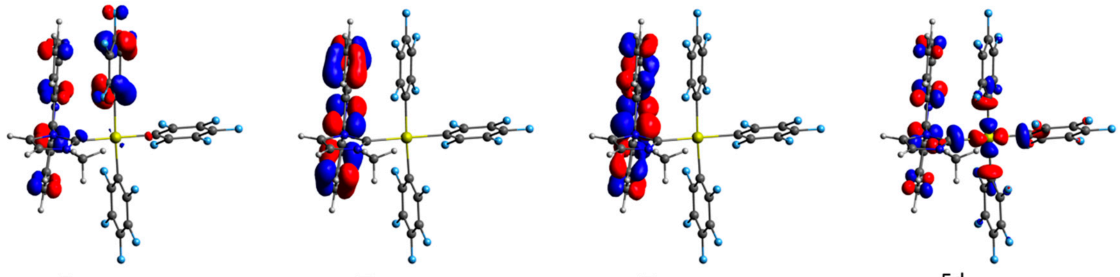

Figure S28. Molecular orbitals involved in the dominant electronic transitions that lead to the studied excited singlet states of complex **3**.

Table S7. Relative entalpies of intermediates and products (kcal·mol^{-1}) of complexes **3**, **4** and **4***.

Reaction to	Int4	Int5	Product
**3**	-45.72	-	-124.10
**4**	-45.72	-66.03	-87.82
**4***	-45.72	-	-113.18

Table S8. Relative free energies of intermediates and products (kcal·mol^{-1}) of complexes **3**, **4** and **4***.

Reaction to	Int4	Int5	Product
**3**	-39.57	-	-116.91
**4**	-39.57	-60.11	-81.03
**4***	-39.57	-	-104.85

Geometries

[$AuCl_3$].

Au 6.92326716174486 4.86554843874823 2.14434762940237

Cl	5.11097287649501	3.67925553226041	1.46780664432671
Cl	8.88706591694928	5.97644476447702	2.38712987335548
Cl	6.19370404481082	5.22401126451433	4.23743585291542

1-(9-acridine)-3-methylimidazol-2-ylidene.

N	2.38144704902653	4.64441505402522	4.75321533992284
N	3.46287247608471	6.45960834725932	5.06241574722664
N	6.59295094896675	9.34405685005261	5.41224750993189
C	3.66408080304361	5.12688934004483	4.74539875261248
C	1.42552048965534	5.61506321086309	5.05993527636204
H	0.35729433130665	5.39999900391942	5.11869310183380
C	2.11207051318060	6.78380959098595	5.25651775924125
H	1.77526768547895	7.78589378179262	5.52417704937648
C	2.06035199742911	3.24739788434451	4.46221119619887
H	0.97212519311366	3.09943164027549	4.53861248459030
H	2.56527119395674	2.58275095505164	5.18231012864688
H	2.38806090298423	2.98456904117421	3.44287680105444
C	4.52020915951656	7.40915865524622	5.17023030922184
C	4.60809981021023	8.46898661530415	4.23706350816026
C	3.71545175867163	8.61932389225233	3.12550534181252
H	2.92244594099429	7.87873999171192	2.97270147161063
C	3.85974682239330	9.68040496213428	2.24892385639548
H	3.17513222326825	9.78339273520683	1.39844927156144
C	4.90059112574148	10.64427498227438	2.43653769630322
H	4.99498622039576	11.47996907918139	1.73236077412667
C	5.78938478624690	10.52172435366717	3.48892303694738
H	6.60222981357608	11.24032893023189	3.64744062063275
C	5.68449313422672	9.43248639943223	4.41842157919349
C	6.50593379495645	8.31762665150377	6.28413050747002
C	7.47824107588739	8.23003074715518	7.33706437742970
H	8.25680661981508	9.00150166259143	7.36705628214454
C	7.42198527325434	7.21144872374336	8.27058810890579
H	8.17097992293371	7.15768955526357	9.07022327658706

C 6.38760119050075 6.22424276509009 8.20790163199378

H 6.35396163742356 5.42929720236910 8.96265541634150

C 5.43283188467640 6.26579186981950 7.20729480751247

H 4.64753826289155 5.50469239401990 7.14925171761952

C 5.46536595819263 7.30069313201221 6.21664526103186

Complex 1.

Au 11.25421510857151 1.35308858255856 4.93102215568022

Cl 11.96736778958248 2.86395962842824 6.60438125622456

Cl 13.32407531921502 0.63067334404823 4.83223695822240

Cl 9.22941195455091 2.31511773089869 4.32862901058909

N 11.01288170053493 0.31763016776743 4.29611466570018

N 11.54868019769796 0.34916558406353 5.85231861688996

N 11.14328111822713 3.27192999001658 8.32442635714378

C 11.15408982002532 0.77770254433790 4.74694515899846

C 11.18005798782211 -0.94055160468647 4.66997119782067

H 10.98108997386001 -1.80110810179848 4.03046297737244

C 11.58978789348005 -0.90267985660278 5.88708120310285

H 11.86199769306661 -1.57344031514074 6.69514084571185

C 10.53986638598865 0.50349847346826 2.96178546981797

H 11.00499029904750 -0.27275134337099 2.33448660412296

H 10.78432690397440 1.49391072806586 2.53989615341715

H 9.44270409127198 0.39501279134365 2.98178002440777

C 11.78903730906476 1.23604155707660 6.70565519210221

C 10.58207251883294 1.49843236945681 6.82051664764323

C 9.87156311470399 0.61310300970000 6.02951419628951

H 10.36889162560405 -0.19290977914582 5.49189998066371

C 8.54317524814293 0.84442422117248 6.09435538159545

H 7.85723767910029 0.20238961037417 5.52966705224038

C 7.97927140397621 1.92847181948614 6.88753583743726

H 6.89161143584006 2.07172099294550 6.88506039681689

C 8.78001395909647 2.78032881924292 7.65632884279105

H 8.37564078185634 3.59742172589872 8.26557701586388

C 10.18650315654417 2.56404593417687 7.64330760812602
C 12.46598694561909 2.89857793268285 8.13355265433187
C 13.52790219300006 3.59243683358488 8.78965638924439
H 13.25951332781184 4.41240902914680 9.46616345202725
C 14.84861569726896 3.23702066117726 8.54836936616422
H 15.65609801919823 3.78406173252144 9.05050702980866
C 15.18897495437643 2.17450711575036 7.63742212881867
H 16.24295787596462 1.93528013481937 7.45233958185021
C 14.19940850796135 1.46531267071537 6.99069887990763
H 14.42208698696400 0.66216643901109 6.26584102397910
C 12.84774302215393 1.81106882680819 7.25427268708564

[AuCl(C_6F_5)$_2$].

Au 9.50807592387705 3.71351435630631 3.62229718986524
Cl 10.12195993645152 2.60900402010816 4.89302833297417
F 8.04206510999870 3.33722669005655 4.69154500704889
F 7.09341201387332 4.19234824804220 3.63131931260538
F 7.39517906307450 5.40764523149739 -0.03130128084150
F 9.70834850083147 4.80574953015494 0.21943820570040
F 10.34525006214383 3.90796339165579 1.80371621172792
F 8.21125805239853 4.86051486803728 4.49758200404934
F 7.53816387539974 6.75262022394332 4.30171965153830
F 8.71037119814673 8.64639304622394 2.98020078209585
F 10.31172085867085 8.26847266798305 1.50307025067421
F 10.90577266584171 5.63950015185280 1.68034907597815
C 9.08968332078347 4.05538709788281 2.96406372100883
C 8.48649079389352 4.09184093687574 3.21198079083216
C 7.88488720233852 4.32853629193791 2.71595416647581
C 8.09508280151170 5.01816509052722 0.93088442364827
C 8.94981311663191 4.53048374481731 1.71841084167719
C 9.39795285217784 4.33674235432618 2.29420085700018
C 9.49762398211855 5.21387973540517 3.16805445842191
C 8.76683533139767 5.40118257112575 3.04914198813499

C 8.48512884464196 6.72019310751397 3.28050089143698

C 8.87531195462874 7.37099611721755 2.99683055911114

C 9.65742765214544 7.26292085586884 2.23111777849914

C 9.95583488702189 6.14881967063924 2.24987478033683

Complex 2.

Au 10.51823660856153 3.73297286002799 3.31987580451121

Cl 11.90409384110546 5.16725472074999 4.66182166777803

F 7.95772933631494 2.45794189034386 3.55733591303301

F 6.13255171028511 2.68733709367426 1.78774903230165

F 6.58707731433560 4.18431546429126 -0.44995782355741

F 8.99713818749722 5.45976880829893 -0.58144793184860

F 10.69740301947626 5.10729987450197 1.42979050465038

F 8.53330751597908 4.28021054442998 4.31803653419011

F 7.49272321136657 6.24747355579227 4.28487410127323

F 8.60773945234434 8.52402009503205 2.88657141901465

F 11.03075261701965 7.51648641346054 1.90118226726060

F 11.72168953301590 5.29888877556200 2.29446390141101

N 10.34141623604210 1.41010164972333 5.00709512794734

N 10.68045077073551 0.92535535775646 3.06011733601537

N 11.19455249703320 2.49919858323694 -0.71445465113296

C 10.50914783774209 1.89008935090792 3.83874729523354

C 10.43094922439322 0.02229240924815 4.98657761188441

H 10.32582793580573 -0.59068310142561 5.88252592602682

C 10.66255809950083 -0.31343283952231 3.67881920710154

H 10.81606619715211 -1.25889059612377 3.15895878656974

C 9.98954115879349 2.23422706627299 6.16444835335676

H 10.23813769879735 1.67408008868001 7.07719069076873

H 10.56662092044528 3.16715790488543 6.11784243096607

H 8.90888129722745 2.47022682172463 6.14606602493055

C 10.75824140151044 1.37094950308869 1.79145866627783

C 9.80881124531773 1.13453094104516 0.81843026713125

C 8.60730936464130 0.41568489945632 1.06441119486876

H 8.41598623876312 0.02060589865295 2.06856835875013

C 7.71424258797670 0.23365759977038 0.01860944610218

H 6.78860715093123 -0.33636668296109 0.17518582907433

C 7.97857050294696 0.79932638181203 -1.27400432918947

H 7.25106657468380 0.64764763916164 -2.08088841411430

C 9.12757779129915 1.53972477652142 -1.51171740862585

H 9.33513237364334 1.99274568435393 -2.48864623955384

C 10.08492184376325 1.74698123960905 -0.47107955848717

C 12.08604032834933 2.69391682924100 0.28553719815120

C 13.28857130665626 3.45924055702062 0.09468317164475

H 13.45982789041524 3.89386448387628 -0.89759339927026

C 14.19864872250040 3.62611854145262 1.13834644500532

H 15.13453183984449 4.20293474173586 0.96209517607728

C 13.94206558338093 3.03452058215406 2.43546915410830

H 14.66256779261358 3.16412739376716 3.25170786977004

C 12.80276681877477 2.28725183830909 2.64893204405488

H 12.56583854284653 1.79735756434120 3.60686646757055

C 11.88808563809566 2.11854917876791 1.58606914751493

C 9.50878793714866 3.73889468694603 2.53064003548046

C 8.31824396480166 3.26833241519134 2.51251757774494

C 7.30466069644536 3.31130586489950 1.60851670000141

C 7.51003941672829 4.05354447509273 0.51104303531719

C 8.71205647360907 4.66557045445719 0.44974503115404

C 9.53049296157656 4.46635376176881 1.51507751501456

C 10.16379205720061 4.63566475301307 3.15020319949828

C 9.32410685733251 5.07938285288031 3.53378246184147

C 8.64281297953445 6.20067125271856 3.58680597727085

C 9.08265205579714 7.27824010453200 2.98241637236351

C 10.17830250827786 6.70855557347463 2.56874551748047

C 10.49636833162167 5.44081142231968 2.76044596028578

[Au(C_6F_5)$_3$].

Au 7.12505516784227 4.98025215887089 2.93245054101415

F 7.11576039141438 6.81830829054013 3.13988392440919

F 4.61697205406711 5.50383056581647 1.52452426094524

F 6.34473792789063 5.42744223601525 1.74878035576525

F 3.01757040461888 3.07832074769449 2.20061882250470

C 6.57277852260154 4.60950629623283 3.09267194832978

F 6.45006149577112 3.10411928141551 3.31205261798608

C 7.68922914629335 5.42627144451120 2.75740539082318

C 5.98015670036085 4.65916505541751 2.83813121223159

F 5.16533898288905 2.32027756857557 3.49334333019176

C 7.86182068188068 6.01871728480031 2.70632089677708

C 5.14590114048256 4.59254132867246 2.56314250926665

C 5.95310139795001 3.72384547839701 3.34708628018578

C 4.14847524623250 3.58493339326144 2.56462000284267

C 5.35284552706585 3.43736298568374 3.18617586388033

F 5.24309849322710 6.58883906259526 3.10892965118245

C 6.34359970532580 3.82474462351007 5.00996955093329

C 5.28494941198151 5.49843919596078 4.03250011786660

C 6.04001405608015 4.68723546187844 4.17116534181885

F 5.40886457003022 3.66530240550782 7.07414151085663

F 7.25212388675895 2.81864654201548 5.03010059942702

F 3.83292488621368 7.00175653058944 4.03220026480977

C 5.48124382128501 4.25021649892975 5.89063914556081

C 4.45079202316336 6.01729930459895 4.60665643620859

C 4.54162130184413 5.27446808514763 5.66352089920502

F 3.73035467777784 5.53048459592870 6.68364371821882

F 9.13244848944993 4.31851294171215 2.36836074760233

F 7.82534813075396 7.82802689140020 2.76006571196572

C 8.79799134891331 5.54684620584195 2.40467701304405

C 8.40959339879348 6.62133497070990 2.49796939272491

C 9.64144345287596 7.12610837931923 1.98757410948002

C 9.56687691843583 5.86975093594294 2.13840022051691

F 10.45035778623643 8.08292544617952 1.65312228181991

F	10.74379885349193	5.19546780632645	1.81494532960459

Complex 3.

C	12.87167026145728	10.71219387585005	16.23297325516208
N	12.61855712346945	11.82891826674182	16.70196442962224
C	13.76894484139485	12.56475014841924	16.72178004614711
C	14.52634320952202	12.36873558187014	17.83668916907384
C	14.02684978801465	11.57199534329465	18.89839767744484
H	13.03068801218729	11.11458530343477	18.80384308546052
C	14.81074487962101	11.42859076653586	20.02285669843348
H	14.43610749301744	10.85703029721257	20.88335315830503
C	16.12520854027101	12.01135438641746	20.07275209102379
H	16.74137856184410	11.85182288524608	20.96674932420087
C	16.62528680625483	12.76745820678543	19.02034553633774
H	17.62346499148158	13.22209929244723	19.05166426829394
C	15.82085767568742	12.99838017575111	17.85821912837184
N	16.25267737780527	13.77787030827754	16.83912462422163
C	15.42605543081911	13.99250726099872	15.78398116586068
C	15.82801207447652	14.84579924949160	14.70507461071833
H	16.82268988339339	15.30596540783951	14.76460552874102
C	14.97151868028108	15.08423729081390	13.63850017316943
H	15.28938351227263	15.74714204956744	12.82337485843173
C	13.67068413644623	14.47749742808671	13.57509915622222
H	13.00666103974594	14.68889149409466	12.72551130165776
C	13.25605718067905	13.62025061478288	14.57281665847705
H	12.28177147964470	13.11342020788246	14.54131208231701
C	14.11765397389081	13.39201569877179	15.68154314456564
C	11.30811858977801	12.02224736523754	17.10083859130135
H	10.93709887231890	12.95351986144558	17.53023626197484
C	10.73047249216665	10.81480467701732	16.81656750858096
H	9.71259378334916	10.44904040220966	16.96121166257227
N	11.74591220222223	10.01594607159094	16.30271956731765
C	11.58780729033030	8.60742351895860	15.97805594794013

H	12.53349141900283	8.23779236266042	15.57272103966213
H	10.75676330518408	8.46855438114813	15.26748125845377
H	11.38920659283825	8.04650241998726	16.90579181063702
Au	14.66734659439435	10.27380398132596	15.63293199002658
C	14.46309005183886	10.65308947980602	14.03710975220887
C	15.07649643315204	11.73585451956141	13.64413576071422
C	15.10714256757608	12.35461659658442	12.42091096783738
F	15.77071714802921	13.50155106428234	12.20374233040136
C	14.47771224754399	11.76740338162803	11.37945847870291
F	14.45402516255605	12.28702150443284	10.14123809795456
C	13.87525147799240	10.60355797964619	11.71064981796893
F	13.25342922863033	9.93504611176072	10.72154191932008
F	15.75927839807666	12.38782511181588	14.61929406016099
C	13.96806269275801	10.11337091401560	12.97555418143027
F	13.35413640547395	8.90035607224240	13.10646750624550
C	15.43133629990617	9.97479908764876	15.38014200250421
C	15.57395811800363	8.81301639855482	15.39015936395445
C	16.50977031165153	7.87732437733140	15.09951490744782
C	17.66667719694453	8.30868856550846	14.63910480557348
F	18.72475345906377	7.57409995931682	14.28430165864308
C	17.46454392460901	9.58182759211132	14.60528840896512
F	18.48968111538393	10.33241820218189	14.12697487952649
F	16.26074070764514	6.57085544355996	15.26321872297576
F	14.46343653434628	8.11801134495087	15.84322957966398
C	16.35061004835604	10.15124367982447	14.98483309697458
F	16.56114240021139	11.41114638246221	14.74115270299582
C	15.15603184700595	10.13210875415198	16.92667766458165
C	16.34111851649663	10.59795710520799	17.08924623406348
F	16.80015552545000	11.30202872271738	16.01171874555206
C	17.16383970091975	10.64888607273068	18.16410472703856
C	16.76085858383492	9.97617507368005	19.26928560014597
C	15.55404701077639	9.39306837915059	19.18494154494856

F 15.06007446363421 8.70467064085794 20.23331997887642

F 17.50384304766204 9.94112686269559 20.38314839183312

F 18.35778764255914 11.25110008739351 18.16748354449416

C 14.91010985928799 9.49439250431513 17.99257720545231

F 13.70893377735623 8.83197544567333 18.07660655011149

1,3-Bis(2,6-diisopropylphenyl)imidazolidin-2-ylidene.

N 2.25403906852152 6.86339152461270 16.35949779708924

N 0.33452699857902 6.71697558860375 17.33378736700583

C 1.61453780792046 7.10778463370066 17.52728809395005

C 1.39044110950290 6.32244020746894 15.26813155693493

H 1.80882191334263 5.38131724269298 14.86783452753559

H 1.32974166357045 7.04951847247005 14.43570817323972

C 0.04929586129983 6.12716628073129 15.99345654497285

H -0.78984267510140 6.65503342459194 15.50555209843190

H -0.23164655868029 5.06208697139137 16.10140981989375

C 3.61438676083698 7.21395089392415 16.09000505505313

C 4.62016480864905 6.22539783223076 16.24245012411644

C 5.95236399716245 6.58430439091692 15.95530571264697

H 6.75259299426306 5.84377355906622 16.07137608441909

C 6.26924843500036 7.88273276635570 15.52522535142210

H 7.31178432868207 8.14589132491230 15.30524124642565

C 5.25937181687181 8.84558516446103 15.37604119477381

H 5.52256705531026 9.85671221943340 15.04261493553171

C 3.91285567262635 8.52852366372697 15.64921614361386

C 4.25829378773688 4.84734722276922 16.78418388757574

H 3.22123256962896 4.63169932888509 16.46410539372728

C 5.15467703382979 3.71690294838296 16.25447629775203

H 5.18886064673353 3.70921104831688 15.14980275427401

H 6.19192894261742 3.80618989433907 16.62729813911016

H 4.76872272633688 2.73847924329905 16.59310042574257

C 4.25184170020774 4.88842977166501 18.32768235021339

H 3.55132888285002 5.66243251562012 18.68905777304770

H	3.93909483430245	3.91335554795279	18.74464949407280
H	5.26233152121126	5.12199692487364	18.71275382428564
C	2.81296225115169	9.57937158395577	15.54690387111538
H	1.85853876086374	9.04271353462408	15.38810519146886
C	3.00133717780408	10.55083200244530	14.36999618151683
H	3.12585659324598	10.00997167204335	13.41427074601320
H	2.11976452156724	11.21102023878514	14.27945877497773
H	3.88425023550780	11.20151079496045	14.51063496596112
C	2.68418110792094	10.33125580029992	16.88919681959648
H	2.45340265720949	9.61867276119310	17.70170639918838
H	3.62691749620770	10.85614370366216	17.13340825046523
H	1.87295355440456	11.08068405132115	16.84102315901731
C	-0.70237068256805	6.79477301336389	18.31391253798781
C	-1.57660403521588	7.91189418780553	18.30111867091414
C	-2.61821331682424	7.95416998580025	19.25012060444920
H	-3.30345104619377	8.81169808763089	19.26304209728075
C	-2.78491673999355	6.91960723810885	20.18308662234298
H	-3.59975486638452	6.96886399255928	20.91659424528921
C	-1.90644449460102	5.82498201712632	20.18420088591796
H	-2.03808708599431	5.02595017754471	20.92478384336221
C	-0.85562335611564	5.73858606307143	19.24861413223204
C	-1.38727054550657	9.05760923820610	17.31459497919267
H	-0.51783189299434	8.80566744485340	16.68092213222464
C	-2.61291476694552	9.22777799073242	16.39613352925203
H	-2.42953545981785	10.02070330735918	15.64799465119486
H	-2.84201802848250	8.29130101700610	15.85590473663966
H	-3.51184305556416	9.51187676781193	16.97471935188547
C	-1.04518628628922	10.36724883286247	18.05213567828959
H	-0.84975953550687	11.18020177428795	17.32889546760031
H	-1.87769317256245	10.68797431577131	18.70595040166132
H	-0.14542558644796	10.24198663827683	18.68006651362402
C	0.12482028143070	4.57331676485727	19.28677559901217

H	0.71744099848867	4.60900452502386	18.35460335113488
C	1.11104689378339	4.76035819274888	20.45814848424423
H	1.86078327280430	3.94824005485027	20.47449114249660
H	1.64481838117204	5.72240838208662	20.36205647953698
H	0.57582631971205	4.75533941694853	21.42638334126471
C	-0.57809371726344	3.20521780979749	19.34703440121184
H	-1.28638749956596	3.08098500651478	18.50788030286261
H	0.16643431258163	2.39009458649252	19.29217563037578
H	-1.14218334883093	3.07594641981425	20.28935366033730

Complex 4*.

Au	2.46678690391894	8.54580095941252	19.83240419828976
F	3.79360468760773	10.27617806828327	19.09714605115088
F	6.24582940317145	10.18152079227687	18.92659995522568
F	7.58500451429882	7.85182083770391	19.59199623238704
F	5.88808809040705	5.93711924092398	20.34792094248390
F	3.46253617634598	6.21635239956429	20.28455844753003
F	1.17462566972504	10.48178734132845	18.69805873414576
F	-0.74920133370239	11.48352993956021	19.83143841357500
F	-1.84000985629750	10.43596442840480	22.02668186072404
F	-0.42156170220182	8.23597463498862	22.91865605847135
F	1.59527910648395	7.49719057270809	21.69862954567912
F	2.77830328848548	11.46859107037843	20.27097393502387
F	3.79709110703284	12.74245290172770	22.63350634214336
F	4.60149897988561	11.33655193542249	24.60149653626317
F	4.53038999171542	8.79188358556570	24.61250816696179
F	3.47874780288728	7.36701303756750	22.33865481784951
C	1.61878321203759	8.99190349587723	20.07267717133963
C	0.91260930518150	9.78301498721319	19.83560293388837
C	-0.18493529944536	10.36152896675673	20.34493775351096
C	-0.74811750674147	9.93654775811891	21.42482556732255
C	-0.06318107331762	8.90801662395643	21.81565417881510
C	0.99841547657433	8.58268145515931	21.09469161391046

C	3.38271614881558	8.21926455126168	19.78676231474997
C	4.27938374124802	9.03082049054275	19.43223434799640
C	5.59461261671247	9.07710942923750	19.31605250779750
C	6.25096740154941	8.00329935043169	19.62289436159550
C	5.36588126790007	7.12108833866853	19.93838776689686
C	4.05350040692655	7.39105814889507	19.93803932615020
C	3.07895432018424	9.35260858704145	21.19763702673129
C	3.20330394364588	10.70465445016483	21.30784296678431
C	3.69138911256015	11.39668171885826	22.43685185011434
C	4.11822120318600	10.70565255102563	23.51918643299020
C	4.03699964164331	9.35533447596029	23.47229433267519
C	3.51254958982816	8.72190124739458	22.32385814430313
N	2.59557814040872	7.47040808016226	17.34759898285294
N	0.66523921506195	7.23027832070602	18.29296855294741
C	1.87227264025373	7.69868479758192	18.40575662196388
C	1.73544971402213	6.93823605439248	16.27092727822765
H	2.25838744992995	6.17960737386946	15.67919295626893
H	1.43237050824056	7.77051048881554	15.60835273828350
C	0.57224688662280	6.39902911731639	17.07558625033697
H	-0.40055366135098	6.51468364797849	16.58669522781912
H	0.71640905168938	5.33824722010981	17.35360058459740
C	3.94253087940273	7.84811539695291	17.06161665395927
C	4.88993497933432	6.83898907993002	16.95226708280918
C	6.20629545436554	7.19567927267823	16.64519192381579
H	6.97397057153519	6.41874170493670	16.56266996778509
C	6.54577995941950	8.53694300089982	16.40255594386440
H	7.58583128937162	8.79698259335125	16.15533971170278
C	5.54211344662149	9.52533206749401	16.40048258359653
H	5.79060751904952	10.55727264490677	16.13199155089456
C	4.21076973629995	9.18459661746026	16.70835361704390
C	4.38215440333683	5.40619643470615	16.95597903279256
H	3.38978936633013	5.43987459232025	17.43934203848246

C	4.25856048325498	4.96873153498620	15.48042038646171
H	3.72316269308355	5.70707711405548	14.86036617400061
H	5.26489335354479	4.84468110935374	15.04308023660196
H	3.72809180291558	4.00342921024843	15.40708903594959
C	5.19036288823144	4.30571782768438	17.63479452771776
H	5.22403575230035	4.40504101475062	18.68555611942478
H	4.72153673791065	3.33047475068106	17.41917817822338
H	6.22750873890774	4.26773415186256	17.25949004265962
C	3.01507915834566	10.10037384223299	16.50901309946560
H	2.18440704426574	9.67814572507822	17.09293075762094
C	2.62865760783297	10.07327653729361	15.01489805399867
H	2.46662808073695	9.04413038728184	14.65145737877037
H	1.69995791108771	10.64750611345531	14.85256455123523
H	3.42800336205639	10.52386791052248	14.40018733058978
C	3.19073489236446	11.54032415574220	16.93595308249700
H	3.40914230390272	11.56510920215901	17.97240854544291
H	3.99895379545524	12.04192271880611	16.37509697163212
H	2.26091300844107	12.10290861255125	16.75473586131272
C	-0.40846155052047	7.24589543960015	19.23114755743919
C	-1.52581184860128	8.01281333876109	18.93004667020757
C	-2.59018522831175	8.01228881279738	19.83641046413322
H	-3.48020701698623	8.61663179382451	19.63045846069594
C	-2.54577534118419	7.21247203180999	20.99067686418165
H	-3.39581392484905	7.22040225485058	21.68840183433440
C	-1.45214335304064	6.35177267966428	21.20813351276412
H	-1.45265066685153	5.67024101780824	22.06498297454953
C	-0.36987193365593	6.34139770459260	20.30799782791578
C	-1.60052065909926	8.62159414723494	17.53823837164887
H	-0.56157369666733	8.65775107363508	17.16627050905678
C	-2.45581066434919	7.66806854705564	16.67550511768410
H	-2.39962506261069	7.95577459674171	15.61123574849319
H	-2.14442188717073	6.61412035794231	16.76670911367404

H -3.51176671870927 7.72403158102206 16.99369975790132

C -2.20892933595312 10.00490464482539 17.33938202272536

H -2.28059697105182 10.21426448290238 16.25841392378385

H -3.22732965597663 10.06979729652300 17.75951916260607

H -1.62473325582747 10.77692227017251 17.76558049821613

C 0.74665322392383 5.31275386129225 20.29943867241786

H 1.56952136078974 5.73885489013645 19.70816098391468

C 1.30476261964938 4.91871977581890 21.64767695600264

H 2.12157641364981 4.19061879807162 21.51955867254500

H 1.68697642516130 5.78303742399608 22.12473576363402

H 0.53833192713653 4.45047450051616 22.29017331954320

C 0.23597806130519 4.04108677081042 19.58959580411609

H -0.16105800510136 4.26246243658398 18.58433450897841

H 1.05656456314997 3.31034050549582 19.48376466976564

H -0.57471132108518 3.57127816781260 20.17423978793016

Complex 4.

Au 1.64799451157650 4.34079586673900 11.37399444472237

F 2.35914343278330 2.41847730790409 10.45725722677546

F 4.33043157127556 1.27578594771075 10.90308648560707

F 6.19359869263513 2.25012390667508 12.78792316722875

F 5.06073141965114 4.63820413842619 13.74939954310583

F 2.96323688005229 5.41426047374183 12.95509104818042

F 0.86526742859288 4.74033469188175 8.98103790002794

F -1.30904434038340 5.48212656561651 8.24379062077452

F -3.34465496554136 6.08099739268858 10.03264912123212

F -2.35809009161672 5.61399105284105 12.59089338628999

F -0.11653674353068 4.80491505182201 12.95514141969379

F 3.17232250994905 3.74578466269408 9.91033664790894

F 4.75179579225210 5.01759103867519 8.68852645418998

F 4.98472128418306 7.77837284628488 9.03938900531733

F 3.09264777605241 8.49865477057670 10.93543151888331

F 1.62832986115024 6.90552497740792 11.94444510443456

F	2.04058615310036	4.20586098623960	13.51299257281060
F	1.46624908266885	2.56376113557175	15.07540622096347
F	0.05624474814430	0.26076232692712	14.32795346095406
F	-0.41021913623674	0.49379585878815	11.59849684065283
F	0.34527993195063	2.38140760809513	10.34151612863582
C	0.61226651975279	4.70366586158364	11.04190229255424
C	0.06689956013713	4.90410959385472	10.06243143017205
C	-1.10874660483965	5.34769136850008	9.57835087164193
C	-2.11072196664044	5.65186568773387	10.34587066382836
C	-1.57454991926227	5.41614522229990	11.50395061935955
C	-0.30416856395848	5.00676195002127	11.63240893783471
C	2.42543379475555	4.01466485124448	11.64408819016522
C	3.01566386058721	3.15361827140202	11.38356257237548
C	4.11267514030838	2.42358181816882	11.58209414957464
C	5.02232835783436	2.79900411047108	12.43215869408484
C	4.42590363750235	3.88834599304913	12.81808723972865
C	3.22398859705069	4.24062892035180	12.33437626892738
C	2.23447170123154	5.13246506732884	11.02147284092675
C	3.01990862110371	5.04171453758871	10.25435280905867
C	3.94925242534881	5.67588450845645	9.55326320171522
C	4.12428457841124	6.95608377949743	9.66049785219782
C	3.21255151501421	7.20268437339225	10.54967358172300
C	2.44742286916724	6.23835146916394	11.09749914253908
C	1.30036325266794	3.50904797976144	11.78516291998203
C	1.35820632578698	3.24873609003486	12.84742770890831
C	1.08622468602552	2.36442944259720	13.79496852679103
C	0.44068179217826	1.27099860155901	13.53244639904114
C	0.26698872773114	1.46541054658165	12.26251148920362
C	0.76004529339679	2.54196134804967	11.61688327927628

Mulliken Charges
[$AuCl_3$].

0 Au: 0.467346

1 Cl: -0.200520

2 Cl: -0.200759

3 Cl: -0.066067

Sum of atomic charges: -0.0000000

1-(9-acridine)-3-methylimidazol-2-ylidene.

0 N : -0.189896

1 N : -0.211785

2 N : -0.145402

3 C : -0.166508

4 C : -0.047219

5 H : 0.135740

6 C : -0.031354

7 H : 0.138341

8 C : -0.116063

9 H : 0.119594

10 H : 0.124386

11 H : 0.125075

12 C : 0.073406

13 C : 0.095853

14 C : -0.140352

15 H : 0.105197

16 C : -0.090739

17 H : 0.111247

18 C : -0.096746

19 H : 0.112948

20 C : -0.095236

21 H : 0.092542

22 C : 0.000711

23 C : 0.000244

24 C : -0.096762

25 H : 0.091749

26 C : -0.095854

27 H : 0.112369

28 C : -0.092714

29 H : 0.110224

30 C : -0.130723

31 H : 0.096401

32 C : 0.101328

Sum of atomic charges: -0.0000000

Complex 1.

0 Au: 0.729651

1 Cl: -0.432756

2 Cl: -0.364098

3 Cl: -0.372972

4 N : 0.094085

5 N : 0.084767

6 N : -0.217005

7 C : -0.294611

8 C : -0.075652

9 H : 0.210873

10 C : -0.149203

11 H : 0.206992

12 C : -0.323304

13 H : 0.174049

14 H : 0.181200

15 H : 0.179462

16 C : 0.164988

17 C : 0.004513

18 C : -0.210043

19 H : 0.175557

20 C : -0.112532

21 H : 0.150019

22 C : -0.104199

23 H :   0.149709

24 C : -0.247953

25 H :   0.128247

26 C :   0.173785

27 C :   0.177348

28 C : -0.249453

29 H :   0.128393

30 C : -0.105971

31 H :   0.149473

32 C : -0.117979

33 H :   0.150902

34 C : -0.246767

35 H :   0.165847

36 C :   0.044640

Sum of atomic charges: -0.0000000

[AuCl(C_6F_5)$_2$].

0 Au: 0.244759

1 Cl: -0.344061

2 F : -0.144117

3 F : -0.146523

4 F : -0.141138

5 F : -0.146989

6 F : -0.145992

7 F : -0.136554

8 F : -0.143859

9 F : -0.140624

10 F : -0.143165

11 F : -0.133502

12 C : -0.154531

13 C :   0.216495

14 C :   0.145512

15 C :   0.153215

16 C :   0.142135

17 C :   0.230239

18 C :   -0.125834

19 C :   0.248768

20 C :   0.129866

21 C :   0.170221

22 C :   0.134758

23 C :   0.230920

Sum of atomic charges: -0.0000000

Complex 2.

0 Au: 0.479405

1 Cl: -0.449629

2 F :   -0.183386

3 F :   -0.167162

4 F :   -0.161305

5 F :   -0.164206

6 F :   -0.179737

7 F :   -0.186183

8 F :   -0.167041

9 F :   -0.163266

10 F :   -0.167481

11 F :   -0.173642

12 N :   0.052074

13 N :   0.089683

14 N :   -0.205378

15 C :   -0.074744

16 C :   -0.070042

17 H :   0.208874

18 C :   -0.176167

19 H :   0.207223

20 C :   -0.312308

21 H :   0.168605

22 H :   0.164504

23 H :   0.174139

24 C :   0.233039

25 C :   0.023566

26 C : -0.297510

27 H :   0.172993

28 C : -0.091457

29 H :   0.148314

30 C : -0.127763

31 H :   0.146198

32 C : -0.213650

33 H :   0.125838

34 C :   0.124857

35 C :   0.154859

36 C : -0.250622

37 H :   0.126338

38 C : -0.102850

39 H :   0.148128

40 C : -0.109292

41 H :   0.145404

42 C : -0.229292

43 H :   0.177962

44 C :   0.021675

45 C : -0.512699

46 C :   0.265858

47 C :   0.161583

48 C :   0.153789

49 C :   0.158356

50 C :   0.266632

51 C : -0.315981

52 C :   0.301303

53 C :   0.114664

54 C : 0.166717

55 C : 0.123252

56 C : 0.246960

Sum of atomic charges: -0.0000000

[Au(C_6F_5)$_3$].

0 Au: 0.419728

1 F : -0.154019

2 F : -0.142513

3 F : -0.153869

4 F : -0.137275

5 C : -0.212746

6 F : -0.153954

7 C : -0.213390

8 C : 0.152282

9 F : -0.142476

10 C : 0.152348

11 C : 0.179330

12 C : 0.151715

13 C : 0.095599

14 C : 0.180356

15 F : -0.130559

16 C : 0.193559

17 C : 0.193194

18 C : -0.244130

19 F : -0.132804

20 F : -0.130296

21 F : -0.132811

22 C : 0.169254

23 C : 0.169441

24 C : 0.124619

25 F : -0.132410

26 F : -0.153432

27 F :   -0.142519

28 C :   0.152698

29 C :   0.179966

30 C :   0.095834

31 C :   0.179029

32 F :   -0.137226

33 F :   -0.142524

Sum of atomic charges: -0.0000000

Complex 3.

0 C :   -0.186955

1 N :   0.053676

2 C :   0.172364

3 C :   0.002590

4 C :   -0.251385

5 H :   0.172532

6 C :   -0.145731

7 H :   0.162937

8 C :   -0.113177

9 H :   0.145806

10 C :   -0.242773

11 H :   0.125435

12 C :   0.185166

13 N :   -0.215785

14 C :   0.183066

15 C :   -0.260733

16 H :   0.127043

17 C :   -0.093440

18 H :   0.147924

19 C :   -0.110699

20 H :   0.148503

21 C :   -0.250801

22 H :   0.169486

23 C :   0.028516

24 C : -0.143162

25 H :   0.195687

26 C : -0.082681

27 H :   0.200016

28 N :   0.086095

29 C : -0.329377

30 H :   0.183477

31 H :   0.173586

32 H :   0.175564

33 Au: 0.616078

34 C : -0.340515

35 C :   0.225151

36 C :   0.108463

37 F : -0.166540

38 C :   0.166274

39 F : -0.164292

40 C :   0.102794

41 F : -0.167810

42 F : -0.178851

43 C :   0.263075

44 F : -0.193340

45 C : -0.390397

46 C :   0.278029

47 C :   0.109490

48 C :   0.159329

49 F : -0.163815

50 C :   0.121289

51 F : -0.167337

52 F : -0.166852

53 F : -0.188563

54 C :   0.234371

55 F : -0.179838

56 C : -0.423429

57 C : 0.284467

58 F : -0.187680

59 C : 0.135879

60 C : 0.136349

61 C : 0.151945

62 F : -0.166867

63 F : -0.163180

64 F : -0.168058

65 C : 0.262078

66 F : -0.190466

Sum of atomic charges: -0.0000000

1,3-Bis(2,6-diisopropylphenyl)imidazolidin-2-ylidene.

0 N : -0.291081

1 N : -0.286389

2 C : -0.033516

3 C : -0.057407

4 H : 0.123158

5 H : 0.120415

6 C : -0.042275

7 H : 0.121703

8 H : 0.120613

9 C : 0.035656

10 C : 0.084563

11 C : -0.175858

12 H : 0.084504

13 C : -0.079163

14 H : 0.096303

15 C : -0.175435

16 H : 0.084939

17 C : 0.091143

18 C : -0.177237

19 H : 0.098207

20 C : -0.232658

21 H : 0.095245

22 H : 0.093823

23 H : 0.094976

24 C : -0.202885

25 H : 0.084698

26 H : 0.093259

27 H : 0.081662

28 C : -0.182840

29 H : 0.098609

30 C : -0.232151

31 H : 0.095058

32 H : 0.094768

33 H : 0.093386

34 C : -0.197795

35 H : 0.078466

36 H : 0.081189

37 H : 0.089660

38 C : 0.038051

39 C : 0.096843

40 C : -0.179919

41 H : 0.090658

42 C : -0.080980

43 H : 0.097042

44 C : -0.177423

45 H : 0.089342

46 C : 0.090389

47 C : -0.184386

48 H : 0.100365

49 C : -0.221962

50 H :   0.095959

51 H :   0.094504

52 H :   0.088988

53 C : -0.221319

54 H :   0.096600

55 H :   0.087935

56 H :   0.097429

57 C : -0.178871

58 H :   0.099274

59 C : -0.208376

60 H :   0.093057

61 H :   0.092499

62 H :   0.083750

63 C : -0.228448

64 H :   0.094736

65 H :   0.094365

66 H :   0.090586

Sum of atomic charges: -0.0000000

Complex 4*.

0 Au: 0.357177

1 F : -0.190872

2 F : -0.168902

3 F : -0.164425

4 F : -0.166850

5 F : -0.191433

6 F : -0.191449

7 F : -0.166813

8 F : -0.164527

9 F : -0.169043

10 F : -0.190545

11 F : -0.184481

12 F : -0.163939

13 F : -0.161463

14 F : -0.163879

15 F : -0.184563

16 C : -0.215789

17 C : 0.207234

18 C : 0.113822

19 C : 0.192383

20 C : 0.093915

21 C : 0.228898

22 C : -0.221539

23 C : 0.230264

24 C : 0.094306

25 C : 0.193029

26 C : 0.110351

27 C : 0.210857

28 C : -0.334976

29 C : 0.244291

30 C : 0.120755

31 C : 0.151752

32 C : 0.120192

33 C : 0.244370

34 N : 0.066781

35 N : 0.064625

36 C : -0.157010

37 C : -0.147224

38 H : 0.171001

39 H : 0.137287

40 C : -0.145281

41 H : 0.170095

42 H : 0.138213

43 C : 0.187928

44 C : 0.059980

45 C : -0.222274

46 H : 0.138185

47 C : -0.195950

48 H : 0.131787

49 C : -0.155422

50 H : 0.130333

51 C : 0.013056

52 C : -0.139786

53 H : 0.113965

54 C : -0.384561

55 H : 0.133361

56 H : 0.134672

57 H : 0.140321

58 C : -0.414689

59 H : 0.140473

60 H : 0.134753

61 H : 0.128160

62 C : -0.111392

63 H : 0.130312

64 C : -0.394553

65 H : 0.127695

66 H : 0.136800

67 H : 0.132108

68 C : -0.419778

69 H : 0.159817

70 H : 0.127766

71 H : 0.129331

72 C : 0.188176

73 C : 0.058635

74 C : -0.220607

75 H : 0.138458

76 C : -0.195744

77 H :   0.131795

78 C :   -0.153695

79 H :   0.133191

80 C :   0.008595

81 C :   -0.139269

82 H :   0.113098

83 C :   -0.384864

84 H :   0.140136

85 H :   0.133713

86 H :   0.134690

87 C :   -0.413850

88 H :   0.134771

89 H :   0.128019

90 H :   0.139879

91 C :   -0.114251

92 H :   0.128739

93 C :   -0.417676

94 H :   0.128491

95 H :   0.159661

96 H :   0.128304

97 C :   -0.394524

98 H :   0.128546

99 H :   0.136564

100 H :   0.132020

Sum of atomic charges: 0.0000000

Complex 4.

0 Au: 0.165454

1 F :   -0.162781

2 F :   -0.156398

3 F :   -0.155850

4 F :   -0.156394

5 F :   -0.162802

6 F : -0.162883

7 F : -0.156391

8 F : -0.155828

9 F : -0.156402

10 F : -0.162811

11 F : -0.162861

12 F : -0.156365

13 F : -0.155846

14 F : -0.156438

15 F : -0.162764

16 F : -0.162865

17 F : -0.156386

18 F : -0.155852

19 F : -0.156380

20 F : -0.162869

21 C : -0.188164

22 C : 0.142283

23 C : 0.164588

24 C : 0.077581

25 C : 0.164637

26 C : 0.142117

27 C : -0.188296

28 C : 0.142387

29 C : 0.164599

30 C : 0.077387

31 C : 0.164837

32 C : 0.141630

33 C : -0.187696

34 C : 0.141934

35 C : 0.164546

36 C : 0.077550

37 C : 0.164621

38 C : 0.142199

39 C : -0.187885

40 C : 0.142067

41 C : 0.164717

42 C : 0.077535

43 C : 0.164654

44 C : 0.141882

Sum of atomic charges: -1.0000000

References

54. Azofra, L.M.; Veenboer, R.M.P.; Falivene, L.; Vummaleti, S.V.C.; Poater, A.; Nolan, S.P.; Cavallo, L. Quantifying Electronic Similarities between NHC-Gold(i) Complexes and Their Isolobal Imidazolium Precursors. *Phys. Chem. Chem. Phys.* **2019**, *21*, 15615–15622, doi:10.1039/c9cp02844g.
55. Pandey, K.K.; Vishwakarma, R.; Bariya, P.K. Quantum Chemical Insight into C-H⋯F Bonding Interactions between Noncovalently Bonded Ion-Pairs in N-Heterocyclic Carbene Complexes of Gold(I) [(NHC∗)2Au]+[PF6]-and Gold(III) [(NHC∗)2AuCl2]+[PF6]-. *J. Organomet. Chem.* **2015**, *795*, 34–39, doi:10.1016/j.jorganchem.2014.11.028.
56. Grimme, S.; Brandenburg, J.G.; Bannwarth, C.; Hansen, A. Consistent Structures and Interactions by Density Functional Theory with Small Atomic Orbital Basis Sets. *J. Chem. Phys.* **2015**, *143*, 54107, doi:10.1063/1.4927476.
57. Weigend, F.; Ahlrichs, R. Balanced Basis Sets of Split Valence, Triple Zeta Valence and Quadruple Zeta Valence Quality for H to Rn: Design and Assessment of Accuracy. *Phys. Chem. Chem. Phys.* **2005**, *7*, 3297–3305, doi:10.1039/b508541a.
58. Andrae, D.; Häußermann, U.; Dolg, M.; Stoll, H.; Preuß, H. Energy-Adjusted Ab Initio Pseudopotentials for the Second and Third Row Transition Elements. *Theor. Chim. Acta* **1990**, *77*, 123–141, doi:10.1007/BF01114537.
59. Kruse, H.; Grimme, S. A Geometrical Correction for the Inter- and Intra-Molecular Basis Set Superposition Error in Hartree-Fock and Density Functional Theory Calculations for Large Systems. *J. Chem. Phys.* **2012**, *136*, 154101, doi:10.1063/1.3700154.
60. Grimme, S.; Antony, J.; Ehrlich, S.; Krieg, H. A Consistent and Accurate Ab Initio Parametrization of Density Functional Dispersion Correction (DFT-D) for the 94 Elements H-Pu. *J. Chem. Phys.* **2010**, *132*, 154104, doi:10.1063/1.3382344.
61. Neese, F. The ORCA Program System. *Wiley Interdiscip. Rev. Comput. Mol. Sci.* **2012**, *2*, 73–78, doi:10.1002/wcms.81.
62. Neese, F. Software Update: The ORCA Program System, Version 4.0. *Wiley Interdiscip. Rev. Comput. Mol. Sci.* **2018**, *8*, 1–6, doi:10.1002/wcms.1327.
63. Barone, V.; Cossi, M. Conductor Solvent Model. *J. Phys. Chem. A* **1998**, *102*, 1995–2001.
64. Hanwell, M.D.; Curtis, D.E.; Lonie, D.C.; Vandermeerschd, T.; Zurek, E.; Hutchison, G.R. Avogadro: An Advanced Semantic Chemical Editor, Visualization, and Analysis Platform. *J. Cheminform.* **2012**, *4*, 1–17, doi:10.1186/1758-2946-4-17.
